# Mass photometry enables label-free tracking and mass measurement of single proteins on lipid bilayers

**DOI:** 10.1038/s41592-021-01261-w

**Published:** 2021-10-04

**Authors:** Eric D. B. Foley, Manish S. Kushwah, Gavin Young, Philipp Kukura

**Affiliations:** 1 Physical and Theoretical Chemistry Laboratory, Department of Chemistry, University of Oxford, Oxford, UK; 2 Present Address: Refeyn Ltd, Oxford, UK

**Keywords:** Membrane proteins, Single-molecule biophysics

## Abstract

The quantification of membrane-associated biomolecular interactions is crucial to our understanding of various cellular processes. State-of-the-art single-molecule approaches rely largely on the addition of fluorescent labels, which complicates the quantification of the involved stoichiometries and dynamics because of low temporal resolution and the inherent limitations associated with labeling efficiency, photoblinking and photobleaching. Here, we demonstrate dynamic mass photometry, a method for label-free imaging, tracking and mass measurement of individual membrane-associated proteins diffusing on supported lipid bilayers. Application of this method to the membrane remodeling GTPase, dynamin-1, reveals heterogeneous mixtures of dimer-based oligomers, oligomer-dependent mobilities, membrane affinities and (dis)association of individual complexes. These capabilities, together with assay-based advances for studying integral membrane proteins, will enable the elucidation of biomolecular mechanisms in and on lipid bilayers.

## Abstract (web summary)

Dynamic mass photometry allows label-free tracking and mass measurement of individual membrane-associated proteins diffusing on supported lipid bilayers. The approach can be used to monitor dynamic (dis)assembly of protein complexes.

## Main

Integral membrane proteins (IMPs) and membrane-associated proteins (MAPs) are essential for a number of cellular processes such as signaling and vesicular trafficking, and this makes them important therapeutic targets^1,2^. Their function often relies on homo- and hetero-oligomerization^3,4^, and this complexity, combined with the need for lipid bilayers, makes it particularly challenging to accurately characterize the stoichiometries and kinetics of the biomolecular interactions underlying IMP and MAP function and regulation. Advances in single-molecule fluorescence-based microscopy methods^5,6^ have enabled in vivo and in vitro investigations of IMP interactions, such as dimerization of G-protein-coupled receptors^7,8^ and nano-clustering^9^, and MAP interactions, such as the coordination of Min proteins during bacterial cell division^10^, and the mechanism of amyloid-β plaque formation on cell membranes, which is associated with Alzheimer’s disease^11^. The main challenges to fluorescence-based methods, however, arise from quantitative uncertainties caused by incomplete labeling of the sample, photochemical and photophysical effects such as photoblinking, photobleaching and quenching, and the distinct labeling required to detect multiple species simultaneously. These limitations have made it challenging to accurately quantify processes such as membrane (un)binding of MAPs and the dynamics and stoichiometries of protein–protein interactions for both MAPs and IMPs. Although numerous approaches aimed at molecular subunit counting exist^12–14^, the analysis and interpretation of the resulting oligomeric distributions is complicated and the number of heterogeneous species that can be detected simultaneously remains limited. Given the critical functional importance of homo- and hetero-oligomeric interactions for membrane-associated processes, there is an urgent need for a quantitative and dynamic approach that is capable of complementing the information accessible from existing methods.

Mass photometry is a label-free method that detects single biomolecules in solution and measures their mass with an overall mass accuracy and resolution of 2% and 20 kDa, respectively^15^. These capabilities enable the quantification of protein–protein interactions in solution with sufficient sensitivity to accurately determine stoichiometry and rate of reactions^16^. As such, mass photometry could be ideally suited to address the shortcomings of existing fluorescence-based techniques for in vitro applications to studying IMPs and MAPs. Existing implementations of mass photometry rely on the stationary binding of individual molecules to a surface, usually a glass coverslip. By averaging images taken before a binding event and subtracting them from averaged images taken after a binding event, the signal due to glass surface roughness is removed and the shot noise is lowered sufficiently to detect individual molecules binding to the surface^17–19^. When molecules remain mobile after binding to the surface, however, the resulting signals are a convolution of the positions of the molecules over the averaged time frame, which makes their detection and quantification difficult. Here, by implementing a new background processing methodology, we show that the capabilities of mass photometry can be extended to in vitro studies of individual protein complexes diffusing on supported lipid bilayers (SLBs).

To explore the suitability of mass photometry to study processes on an SLB, we chose the 100 kDa MAP wild-type dynamin-1 (WT) on a 60–40 1,2-dioleoyl-*sn*-glycero-3-phosphocholine (DOPC)–1,2-dioleoyl-*sn*-glycero-3-phospho-l-serine (DOPS) bilayer, in line with previous in vitro investigations^20–28^. Dynamin is a multi-domain, large GTPase that can catalyze membrane fission during clathrin-mediated endocytosis^29^. Its role in membrane constriction and fission relies on its (dis)assembly on lipid bilayers, with our current understanding of the underlying molecular mechanisms of dynamin polymerization based predominantly on structural information and bulk behavior^30^. Single-molecule fluorescence studies on dynamin have struggled to resolve oligomeric distributions^31,32^, making dynamin a particularly attractive system for mass photometry. By applying an alternative background-removal approach to mass photometry, referred to as dynamic mass photometry, to images of dynamin diffusing on an SLB, we achieve sufficient sensitivity to track individual dynamin oligomers while simultaneously measuring their mass. Due to the label-free nature of mass photometry, the observation time of individual molecules is limited only by the time in which they remain bound to the SLB and/or in the field of view. Furthermore, we achieve sub-50 kDa mass resolution while also enabling quantification of oligomer-specific diffusion coefficients and membrane affinities, making dynamic mass photometry a powerful method for studying membrane-associated biomolecular processes.

## Results

### Label-free imaging of mobile dynamin oligomers

**Fig. 1:**
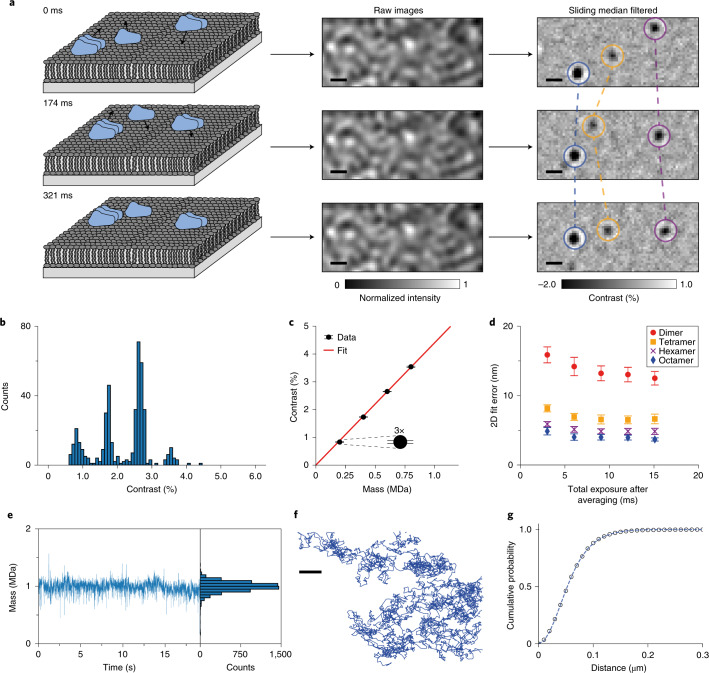
Principle and performance of dynamic mass photometry. **a**, Schematic diagram of dynamic mass photometry of protein complexes diffusing on an SLB. The images were acquired at 331 Hz and processed with a sliding median filter, which showed individual protein complexes on the bilayer as diffraction-limited spots. This procedure consistently gave similar results (*n* > 30). **b**, Histogram of mean trajectory contrasts detected in a dynamic mass photometry movie (*n* = 1 movie, 4 min) of WT diffusing on an SLB (considering only trajectories of at least 151 ms in length; *n* = 425 trajectories). **c**, Contrast–mass calibration curve of the dynamic mass photometry measurement shown in **b** (*n* = 1 dynamic mass photometry movie, 4 min) yielding a contrast to mass ratio of 4.40 % MDa^−1^. Error bars represent the mean contrast ± s.e.m. of each oligomeric species (*n*_dimer_ = 34, *n*_tetramer_ = 85, *n*_hexamer_ = 184, *n*_octamer_ = 23 trajectories). **d**, 2D localization error of our PSF-fitting procedure of WT dimers, tetramers, hexamers and octamers plotted as a function of effective exposure time. Data are given as the mean localization errors in 2D ± the combined s.d. of the mean errors in x and y of particle trajectories detected during the dynamic mass photometry movie in **b** (*n* = 1 movie, 4 min), processed with different amounts of frame averaging (*n*_dimer_ = 34, 51, 60, 52, 73; *n*_tetramer_ = 82, 102, 98, 97, 94; *n*_hexamer_ = 177, 229, 224, 208, 173; *n*_octamer_ = 22, 29, 37, 38, 33 trajectories for total exposure times of 3.0, 6.0, 9.1, 12.1 and 15.1 ms, respectively). **e**, Mass trace and histogram of a WT decamer trajectory (*n* = 6,061 frames). **f**, Corresponding particle trajectory. **g**, Corresponding cumulative probability of particle displacements during 1 frame (*t* = 3 ms) and the fits to a two-component model (equation 4). Scale bars, 500 nm. Source data

**Extended Data Fig. 1:**
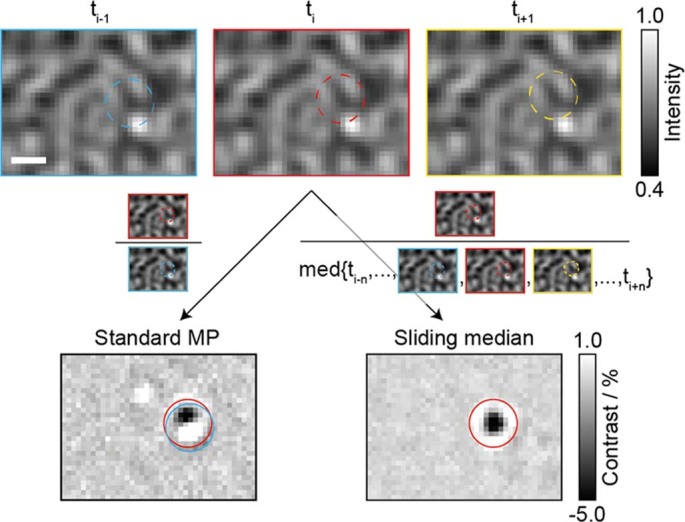
Background subtraction in standard mass photometry (MP) vs dynamic MP. Zoom on three consecutive raw images from a dynamic MP movie of WT dynamin in contact with an SLB containing a particle diffusing upwards through the image (top row), which is masked by the large signal from the glass surface roughness. In standard MP (left), images are divided by preceding images to remove the large static signal due to surface roughness. This background subtraction relies on stationary binding of small particles to the surface to visualise them. When particles bind to and diffuse on the surface, the background subtraction used in standard MP results in a signal that is a convolution of the particle’s position at t_i-1_ and t_i_, (blue and red, respectively), which is challenging to reliably detect and quantify. A sliding median filter (right), that is subtracting each image’s temporal median background obtained from a defined window of images around the image of interest, reveals signals of only the particles in the image of interest (t_i_, red). For further explanation see ‘Data processing’ in the Methods section. Scale bar = 500 nm. The effect illustrated in this figure was reproducible in all measurements shown in this study (n > 30).

Raw mass photometry images of dynamin on an SLB exhibited an optical background caused by the roughness of the microscope coverslip (Fig. 1a; raw images). By implementing a sliding median background subtraction^33^, we obtained a nearly shot noise-limited imaging background, revealing diffraction-limited features arising from individual WT complexes diffusing on the SLB (Extended Data Fig. 1 and Supplementary Video 1). The sliding median background subtraction involves estimating the static imaging background from the temporal median of a series of frames around each frame of interest (see Methods). Importantly, this approach avoids the convolution of scattering contrast and particle motion inherent in the background subtraction used in standard mass photometry, and reduces the imaging background at equivalent imaging speeds due to the larger number of frames contributing to the background image (Extended Data Fig. 1 and Supplementary Fig. 1).

**Extended Data Fig. 2:**
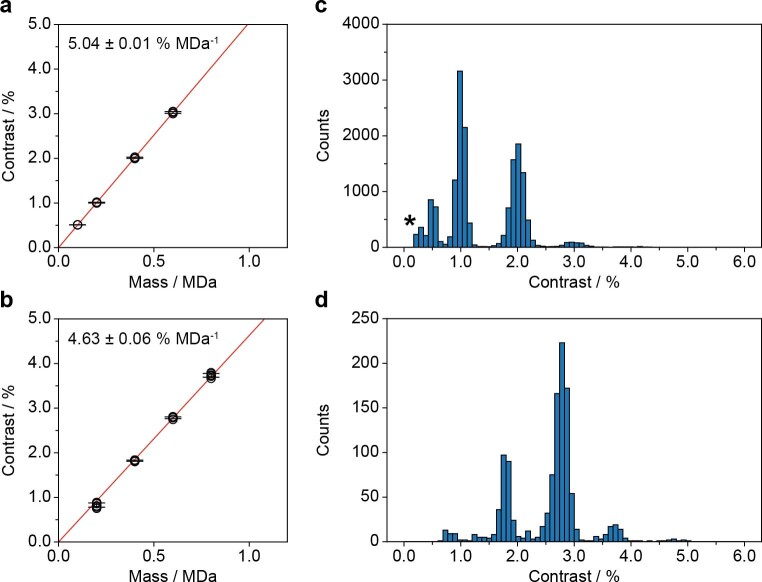
Comparison between WT in solution and on the SLB. **(a)** Mean contrast vs mass calibrations obtained from n = 3 standard MP measurements of WT (40 nM, n_monomer_ = 832, n_dimer_ = 2637, n_tetramer_ = 2404 and n_hexamer_ = 263 particles) and **(b)** from n = 6 independent 2 min dynamic MP movies (using only trajectories that lasted at least 20 frames) of 20 nM WT on an SLB (n_dimer_ = 227, n_tetramer_ = 1079, n_hexamer_ = 2482 and n_octamer_ = 311 trajectories) acquired on the same day. The error bars in (a) and (b) represent the mean ± s.d. of the contrast of each oligomeric species from the repeat measurements. In some cases the standard deviation was less than 1% causing the contrast error bars to overlap. **(c)** Oligomeric distribution of WT (100 nM) in HKS-100 buffer measured by standard MP (n = 4 combined measurements with a total of n = 16794 particles). The peaks represent WT monomer (0.5%), dimer (1.0%), tetramer (2.0%) and hexamer (3.0%). **(d)** Oligomeric distribution of 10 nM WT in HKS-100 buffer diffusing on an SLB obtained from n = 2 combined sets of 3 min dynamic MP movies considering only trajectories that lasted at least 50 frames (n = 1187 trajectories). The contrast measured in dynamic MP movies was consistently ~8% lower than that measured in standard MP. This effect is likely a result of particle motion during image acquisition, which results in motion blurring of the PSF (Supplementary Fig. 3 and Extended Data Fig. 3). This effect increased as we increased frame averaging in dynamic MP movies (Extended Data Fig. 4). The standard MP measurements were acquired at 331 Hz and then processed at a final integration time ~24 ms (effective frame rate ~41 Hz), which enabled the detection of WT monomer. *Peak due to background noise. Source data

For the chosen system, the detected particles exhibited clearly differing signal intensities (Fig. 1a, filtered images, and Supplementary Video 1). Filtering for trajectories that remained bound to the SLB for at least 50 frames, corresponding to a residence time of 151 ms (Supplementary Fig. 2), and plotting the mean contrast of the remaining 425 trajectories revealed a contrast distribution with equally spaced peaks, as expected for different oligomeric states (Fig. 1b). The contrast values of these particles increased linearly with mass (Fig. 1c) and matched well with the expected contrasts of WT dimers, tetramers, hexamers and octamers based on standard mass photometry measurements (Extended Data Fig. 2a,b and Supplementary Table 1), demonstrating that dynamic mass photometry can simultaneously image, track and measure the mass of diffusing biomolecular complexes on SLBs. Additionally, the oligomeric distribution of WT on the SLB displayed a shift to higher oligomeric states compared with the solution distribution measured using standard mass photometry (Extended Data Fig. 2c,d).

### Localization precision and effect of imaging speed

**Extended Data Fig. 3:**
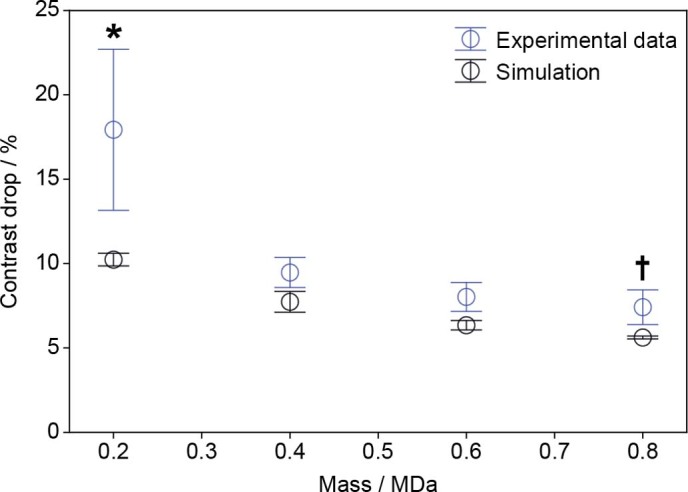
Contrast decrease in dynamic MP vs standard MP. Drop in contrast of different WT oligomers (dimer, 0.2 MDa; tetramer, 0.4 MDa; hexamer, 0.6 MDa; octamer, 0.8 MDa) when comparing dynamic MP measurements to standard MP measurements (blue circles) and drop in contrast observed in simulated dynamic MP movies (Supplementary Fig. 3). For experimental data the contrast drop represents the mean reduction in the average contrast of each oligomeric species measured in n = 6 dynamic MP measurements (2 min each, considering only trajectories that lasted at least 20 frames resulting in a total of n_dimer_ = 227, n_tetramer_ = 1079, n_hexamer_ = 2482 and n_octamer_ = 311 trajectories) compared to the average contrast measured in n = 3 standard MP measurements (Supplementary Table 1, total of n_dimer_ = 2637, n_tetramer_ = 2404 and n_hexamer_ = 263 particles). For simulated data, the contrast drop represents the decrease in contrast of each species detected in n = 3 processed simulated movies compared to the contrast value that was used to simulate the point spread functions onto the raw images (1.00-4.00% for dimer-octamer, Supplementary Table 2, n_dimer_ = 1780, n_tetramer_ = 2302, n_hexamer_ = 2618 and n_octamer_ = 2745 trajectories). Data is presented as mean values ± s.d.. *In dynamic MP movies the contrast of dimer particles partially overlaps with that of background signal, which most likely causes underestimation of the dimer contrast and an exaggerated decrease in contrast compared to standard MP measurements. ^**†**^WT octamer was not detected in standard MP measurement and the contrast was extrapolated using the contrast vs mass calibration. Source data

**Extended Data Fig. 4:**
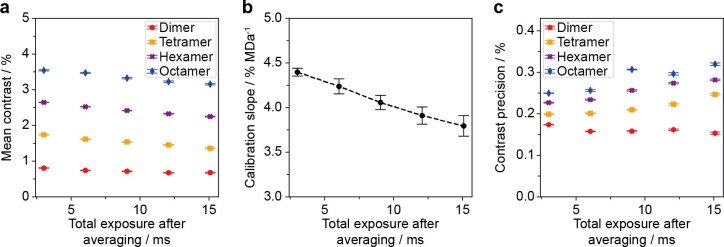
Effect of frame averaging on particle contrast. **(a)** Mean contrast of WT dimer (red circles), tetramer (orange squares), hexamer (purple crosses) and octamer (blue diamonds) trajectories, **(b)** mean contrast vs mass calibration slope obtained from the dynamic MP movie in (a) vs single frame length after averaging and **(c)** contrast precision of our PSF-fitting procedure for each oligomer (same symbols as in (a)) all plotted vs total exposure time of 1 frame after averaging. These trends are most likely a result of particle motion during image acquisition, which becomes more pronounced as more raw images are averaged together and the frame length increases. The plots were obtained from the same movie of WT used in Figs. 1b-d and 2a (n = 1 movie (4 min) of 20 nM WT) with additional frame averaging of 1, 2, 3, 4 and 5 frames, which corresponds to frame lengths of 3.02, 6.04, 9.05, 12.07 and 15.09 ms or frame rates of 331, 166, 110, 83 and 66 Hz, respectively (see Supplementary Information). The data in (a) and (c) are presented as mean values ± s.e.m. for each oligomeric species. The data in (b) is presented as mean values ± s.d.. For these plots n_dimer_ = 34, 51, 60, 52, 73, n_tetramer_ = 82, 102, 98, 97, 94; n_hexamer_ = 177, 229, 224, 208, 173; n_octamer_ = 22, 29, 37, 38, 33 trajectories for total exposure times of 3.0, 6.0, 9.1, 12.1, 15.1 ms, respectively. Source data

Localization precision and imaging speed are key performance parameters for single-particle tracking and contribute decisively to the type of information that can be extracted from individual trajectories. The nature of the sliding median background subtraction prevented the assessment of localization precision by repeated measurement of the location of surface-immobilized particles, as is commonly done in fluorescence-based methods^34^. Nevertheless, we could estimate the localization precision by extracting the error of our point spread function (PSF)-fitting procedure (Fig. 1d). At the imaging speed of 331 Hz (3 ms total exposure time), the fit error for WT dimers (200 kDa particles) was 16 nm, which compares well with the localization precision in single-molecule fluorescence imaging at similar speeds (18 nm) (ref. ^35^), and improved with increasing mass (8, 6 and 5 nm for WT tetramers, hexamers and octamers, respectively). Localization errors improved by up to 20% when lowering the effective imaging speed to 110 Hz, beyond which there was no further improvement. On average, we found that the slope of the contrast–mass calibration curve in dynamic mass photometry was 8% lower than in standard mass photometry, in which particles are stationary. This drop in contrast matched well with the contrast decrease expected from particle movement during image acquisition, that is, motion blur (Extended Data Fig. 3 and Supplementary Tables 1–3). This trend became more pronounced as we lowered the effective imaging speed from 331 Hz to 66 Hz, resulting in a drop in contrast precision of 20% and a further 15% decrease in particle contrast (Extended Data Fig. 4). As such, we attribute these effects to motion blurring of the PSFs, which results in decreased particle contrast and diminished improvements in localization precision at lower imaging speeds. We thus chose to image at 331 Hz to minimize the effects of motion blurring. As a result, however, we were unable to detect WT monomers on the SLB, and in some cases it was difficult to distinguish WT dimers from background noise. We therefore excluded dimeric particles from the mobility and membrane affinity analysis.

### Quantifying oligomer-specific mobility and membrane affinity

Given that dynamic mass photometry is not subject to photobleaching, the time limit on observing particle trajectories is in principle determined only by how long the particles remain bound to the membrane and/or within the field of view. The longest trajectory we could identify lasted more than 6,000 frames with robust localization precision and mass measurement (Fig. 1e,f and Supplementary Video 2). From these data, we could compute the diffusion coefficient by fitting multiple-mobility models to the cumulative probability distribution of particle displacement (Supplementary Fig. 4a) during a defined lag time, *t* (equations 3–5) (ref. ^5^). For the WT decamer particle in Fig. 1e,f, a two-component fit was determined to be the most suitable (see Methods), and produced major and minor diffusion coefficients of D_1_ = 0.58 μm^2^ s^−1^ and D_2_ = 0.22 μm^2^ s^−1^ with relative weightings of 0.56 and 0.44, respectively, using *t* = 3 ms (Fig. 1g).

**Fig. 2:**
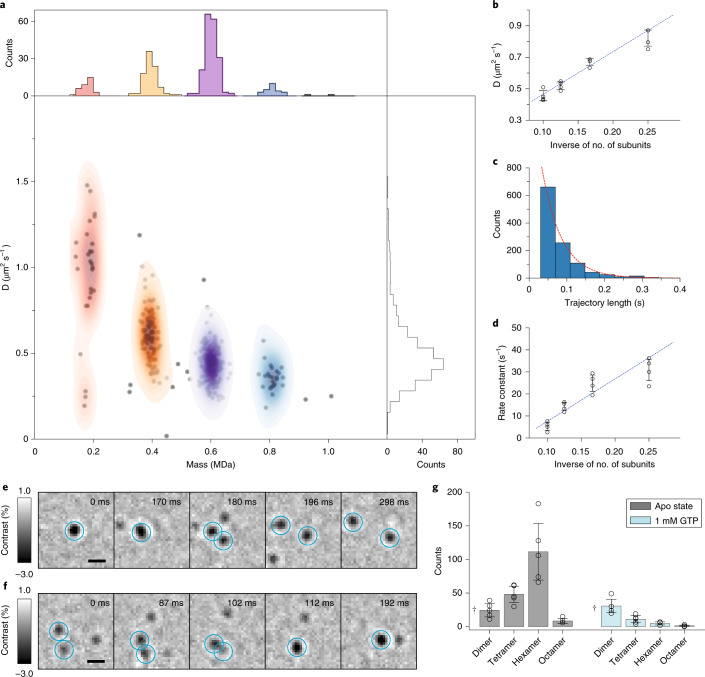
Oligomeric properties and dynamics of dynamin diffusing on an SLB. **a**, Major diffusion components versus mean trajectory mass for a dynamic mass photometry movie (*n* = 1 movie, 4 min) of WT (20 nM); *n* = 333 trajectories. **b**, Major diffusion components of each oligomeric species of ΔPRD (10–20 nM) determined from *n* = 4 replicate dynamic mass photometry measurements (4–5 min each with a total of *n*_tetramer_ = 213, *n*_hexamer_ = 937, *n*_octamer_ = 330 and *n*_decamer_ = 83 trajectories) versus the inverse of the number of oligomeric subunits, and a corresponding weighted linear fit (blue dashed line). Error bars represent the mean ± s.d. **c**, Histogram of SLB residence times of ΔPRD hexamer trajectories from one of the dynamic mass photometry movies with a fit to a 1-component exponential distribution (appropriately scaled here for display) yielding a dissociation rate constant of 19.4 s^−1^. *n*_hexamer_ = 1,123 trajectories. **d**, Dissociation rate constants of each oligomeric species of ΔPRD versus the inverse of the number of oligomeric subunits from *n* = 4 independent replicate dynamic mass photometry measurements (10–20 nM, 4–5 min each with a total of *n*_tetramer_ = 574, *n*_hexamer_ = 5,291, *n*_octamer_ = 925 and *n*_decamer_ = 69 trajectories) and a corresponding weighted linear fit (blue dashed line). Error bars represent the mean ± s.d. **e**,**f**, Examples of a dissociation event (**e**) and an association event (**f**). These events were extremely rare (<1 in 1,000 trajectories). **g**, Effect of GTP addition on the oligomeric distribution of 10–20 nM WT (*n* = 5 independent replicates of 1 min dynamic mass photometry movies before and after GTP addition). Data are given as mean ± s.d. ^†^WT dimer partially overlapped with background noise and could not always be reliably identified. Scale bars, 500 nm. Source data

**Extended Data Fig. 5:**
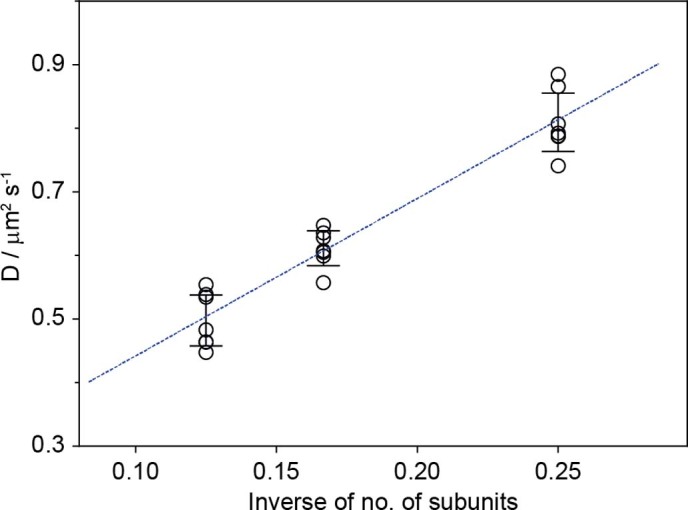
Diffusion coefficient vs inverse of number of subunits of WT oligomers. Mean diffusion coefficients of each oligomeric species of WT from n = 7 independent repeat measurements (n_tetramer_ = 498, n_hexamer_ = 1326, n_octamer_ = 156; data shown in Supplementary Fig. 6-7) vs the inverse of the number of subunits of each oligomeric species and a corresponding weighted linear fit (blue dashed line). Error bars are presented as mean values ± s.d.. Source data

**Extended Data Fig. 6:**
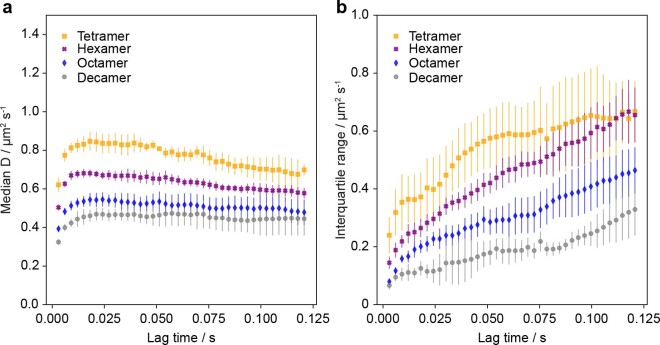
Effect of lag time on calculated diffusion coefficients. **(a)** Average median diffusion coefficient and **(b)** corresponding interquartile range vs chosen lag time (*t*) for each oligomeric species from n = 4 independent ΔPRD measurements (n_tetramer _= 213, n_tetramer_ = 937, n_hexamer_ = 330, n_decamer = _83; trajectories data shown in Supplementary Fig. 8-9). As the distribution of diffusion coefficients broadened significantly as the lag time increased, the diffusion coefficient of each oligomer was determined by taking the median of the distribution instead of Gaussian fitting. Each data point represents the mean diffusion coefficient from the median values determined from four repeats of ΔPRD measurements and the error bars are presented as mean values ± s.d.. Source data

Applying this approach to 20 nM WT, a concentration chosen to achieve a suitable particle density for single-molecule measurements, we were able to measure the diffusion coefficients of different oligomeric species, resulting in a mass resolution of <50 kDa (Fig. 2a). More than 95% of species exhibited only one type of diffusive behavior (Supplementary Figs. 4b and 5), as expected for simple Brownian motion. Further repeat measurements with WT (Extended Data Fig. 5, Supplementary Figs. 6, 7 and Supplementary Table 4) and its 90 kDa mutant, ΔPRD (Fig. 2b, Supplementary Figs. 8,9 and Supplementary Table 5), which is more oligomerization prone than WT^23^, revealed a reproducible inverse proportionality of the diffusion coefficient with the number of oligomeric subunits. Given that the diffusion of membrane-bound proteins has been reported to depend primarily on their contact area with the SLB and the number of bound lipids^36–38^, our results suggest that the contact between the SLB and the oligomers of WT and ΔPRD in the range observed here increases linearly with oligomeric state. Additionally, we observed an increase in calculated diffusion coefficients of all oligomeric species when increasing the lag time from 3 to 12 ms (Extended Data Fig. 6), most probably caused by the dynamic error originating from a combination of the relatively fast particle motion with nanometer localization precision^39^. At longer lag times there was little change in the diffusion coefficients, again confirming that dynamin undergoes Brownian motion on the timescales relevant to this study.

**Extended Data Fig. 7:**
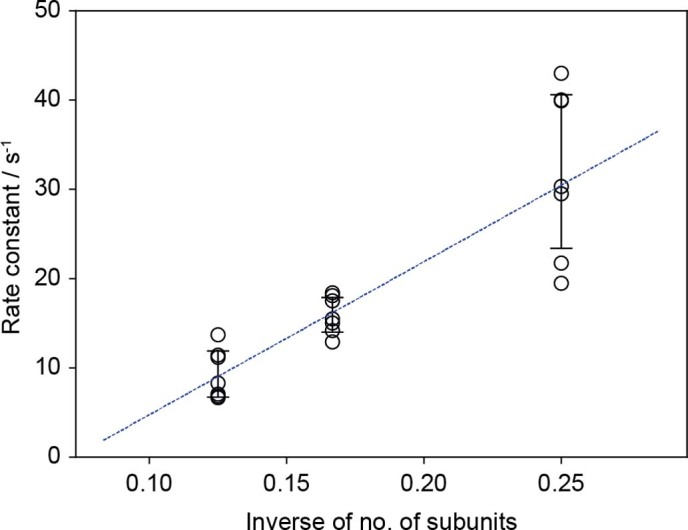
Dissociation constant vs inverse of number of subunits of WT oligomers. Mean dissociation constant from the SLB of each oligomeric species of WT determined from n = 7 independent repeat measurements (n_tetramer_ = 2263, n_hexamer_ = 3264, n_octamer_ = 203 trajectories; data shown in Supplementary Fig. 11) vs the inverse of the number of subunits of each oligomeric species and a corresponding weighted linear fit (blue dashed line). Error bars are presented as mean values ± s.d.. Source data

**Extended Data Fig. 8:**
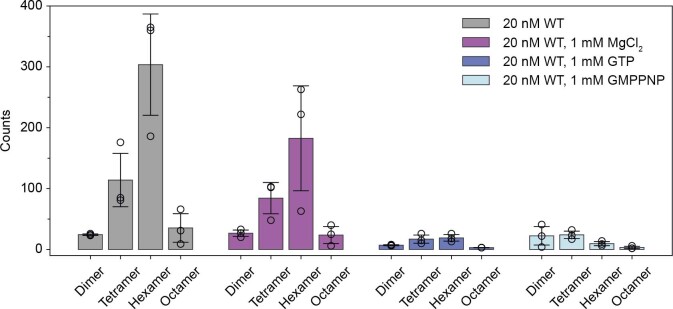
Effect of GTP and GMPPNP on the oligomeric distribution of WT. Average oligomeric distribution of 20 nM WT in contact with an SLB in its apo-state, with 1 mM GTP, with 1 mM of GMPNP (non-hydrolysable GTP analogue) and with 1 mM MgCl_2_ instead of 2 mM MgCl_2_. The 1 mM MgCl_2_ measurement was included as a control in case the presence of GTP/GMPNP results in a reduction in free Mg^2 + ^, which could potentially affect the membrane affinity of WT dynamin oligomers. Each bar plot was generated by taking the mean oligomeric counts from n = 3 repeat measurements (1 min dynamic MP movie each). Error bars are presented as mean values ± s.d.. Source data

We were also able to quantify the residence times of dynamin species bound to the SLB (a measure of the affinity of dynamin to the SLB) and their dependence on oligomeric state. We found that the distribution of residence times of both the WT and ΔPRD oligomers was well described by an exponential model (Fig. 2c), as expected for a first-order process, from which we could extract the dissociation rate for each oligomeric species (Supplementary Figs. 10 and 11). The majority of trajectories lasted less than 300 ms and, similarly to the diffusive behavior, the dissociation constants were inversely proportional to the number of subunits (Fig. 2d, Extended Data Fig. 7 and Supplementary Tables 4 and 5), that is, to oligomeric mass, suggesting that SLB contact increases linearly with oligomeric state. Additionally, we found instances of dynamin oligomers dissociating into subunits on the SLB (Fig. 2e and Supplementary Video 3) and vice versa (Fig. 2f and Supplementary Video 4). Such events were rarely observed in our experiments (in less than 0.1% of trajectories), suggesting that they tend to happen on timescales longer than the SLB residence times for dynamin in its apo state (<200 ms on average). Upon the addition of GTP, which is essential to dynamin function and disassembly from the membrane, we found that the overall particle density of WT immediately decreased (Fig. 2g and Supplementary Fig. 12). Addition of the non-hydrolysable analog 5′-guanylyl imidodiphosphate (GMPPNP) had the same effect (Extended Data Fig. 8), suggesting that GTP binding triggers a decrease in the SLB affinity of dynamin oligomers.

## Discussion

We have demonstrated accurate and resolved mass measurement of proteins diffusing on supported lipid bilayers at the single-molecule level without extrinsic labels. We were able to quantify key parameters such as oligomeric distribution, residence times (that is, membrane affinity) and diffusion coefficients, and observed (dis)association events all at a temporal resolution of 3 ms. The ability to quantify mass, diffusion coefficients and membrane affinities also allowed us to validate our observation of dynamin dimers, tetramers, hexamers, octamers and decamers on the SLB, suggesting that dynamin oligomerization proceeds by dimer addition, in agreement with fluorescence and structural-based studies^23,24,31,40^, instead of being based on exclusively tetrameric subunits, which is the predominant species in solution^41^. As such, our results suggest that single-molecule studies could further our understanding of the importance of tetrameric particles in the dynamics of dynamin polymerization. Our observation of decreased membrane affinity caused by GTP binding is intriguing considering that dynamin can tubulate membranes effectively both in the apo and GTP-bound states^25,40^. A possible explanation for this observation could be that GTP-binding-induced conformational change^42,43^ imparts curvature sensitivity to dynamin oligomers, leading to their dissociation from flat, supported bilayers, although further experiments on curved membranes are necessary to validate this hypothesis. To examine the mechanism of dynamin assembly in more detail, comparison of the oligomeric (dis)assembly of dynamin-1 and of the mutants that affect dynamin assembly^44^, along with disease-associated mutants^45–47^, on SLBs containing phosphatidylinositol 4,5-bisphosphate (PIP2), which specifically recruits dynamin^21,48^, could provide extremely valuable insights. Similarly, these experiments would benefit from the quantification of oligomeric distributions in a more physiologically relevant setting such as curved membranes, although we would expect the kinetics, not the mechanism of the assembly, to be affected given that the rates of dynamin polymerization are controlled by membrane curvature^21^.

**Extended Data Fig. 9:**
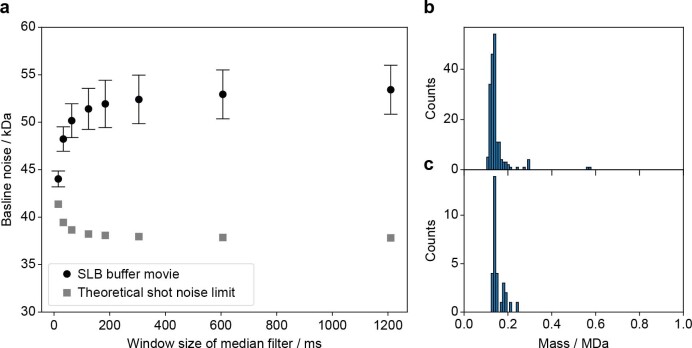
Analysis of background noise in dynamic MP movies. **(a)** Mean baseline noise of the movie of HKS-100 buffer in contact with an SLB (black circles) shown in Supplementary Fig. 1 vs window size of the sliding median filter used to process the movie and the corresponding theoretical shot noise limit (grey squares). The error bars represent the mean baseline noise ± standard deviation across n = 4599 recorded frames. The inverse trend in baseline noise of the buffer movie compared to the theoretical shot noise is a result of particle-like background features in dynamic MP movies, which are subtracted out by the sliding median filter at short window sizes (for example 20-100 ms) but not at longer window sizes (> 200 ms). **(b-c)** Mass histogram of n = 182 and n = 30 trajectories, respectively, detected in a single SLB buffer movie processed with the same settings described in the Methods section, filtered for trajectories with a minimum length of 10 and 50 frames, respectively. The detected background features have contrasts corresponding to ~150 kDa, which prevented the detection of ΔPRD dimer and made reliable detection of WT dimer challenging. Source data

The potential shortcomings of our approach include a limitation on the concentration range, that is, to reliably resolve and quantify single molecules the particle densities on the SLB should be <1 μm^−2^. Currently, the need for SLBs excludes studies of integral membrane proteins due to the unavoidable interaction with the supporting glass substrate. In the future, we expect that the use of cushioned, suspended or tethered lipid bilayers^49^ will expand the demonstrated capabilities to include integral membrane proteins. This advance will enable quantitative and stoichiometric studies of homo- and heterotypic interactions of IMPs and MAPs with each other and with other soluble proteins, and the effect of therapeutics in near native environments. Currently, the detection limit and the mass resolution of dynamic mass photometry are limited by background fluctuations and particle-like features present on the SLB (Extended Data Fig. 9). As such, further improvements in SLB formation will result in improved mass sensitivity and resolution, and provide access to the majority of protein–protein interactions in and on lipid membranes. More generally, we expect that single-protein detection and mass measurement on lipid bilayers will prove powerful for the specific, label-free detection of biomolecules in complex mixtures, and has the potential for further improvements in the capabilities of mass photometry through prolonged observation of individual molecules.

## Methods

### Stocks, reagents, and instruments

For the list of stocks, reagents and instruments that were used in this study and their suppliers, as well as how the buffer stocks were prepared, please refer to the Materials section in the Supplementary Information.

### Protein expression and purification

For details on the expression of WT and ΔPRD, purification, size-exclusion chromatography and storage conditions, please refer to the Supplementary Information.

### Supported lipid bilayer preparation

Liposomes were prepared by dissolving DOPC (Avanti Polar Lipids; 850375 P) and DOPS (sodium salt) (Avanti Polar Lipids; 840035 P) in chloroform and mixing them in a clean glass tube at a 60–40 molar ratio (500 μM total lipid concentration). The mixture was then dried under a constant nitrogen stream via rotary evaporation, and further dried under vacuum for 1 h at room temperature (22–23 °C). A total of 500 μl HKS-150 (20 mM HEPES, pH 7.4, 150 mM KCl; see buffer preparation in the Supplementary Information) was added to the dried lipids and the mixture was covered with parafilm, incubated at 50 °C in a water bath for 1 h, briefly vortexed, and stored overnight at room temperature. The resulting liposome mixture was transferred into a 1.5 ml Eppendorf tube and kept in ice-water and sonicated using a 3 mm probe at 25% amplitude with a 1 s on–3 s off sonication cycle (Sonics & Materials) for 10 min (that is, 40 min in total). Sonicated liposomes were then spun at 20,000 ×*g* for 30 min at 4 °C and the supernatant was collected in an Eppendorf tube, stored at 4 °C, and used within 3 d.

To prepare fluid SLBs, glass coverslips (24 × 50 mm, Menzel Gläser, VWR 630-2603) were cleaned by sonication in Milli-Q water for 5 min, isopropanol for 5 min, and Milli-Q water again for 5 min in an ultrasonic bath, dried using a nitrogen stream and stored in a dry place until use. Before SLB preparation, coverslips were treated with oxygen plasma for at least 8 min using a Zepto plasma cleaner (Diener eletronic) at maximum power. Silicone gaskets (6 mm × 1 mm, GBL103280, Grace Bio-Labs) were rinsed sequentially with Milli-Q water, isopropanol and Milli-Q water, dried under a nitrogen stream, and placed on the freshly plasma-cleaned coverslips along with 30 μl freshly reconstituted HKS-150 containing 1.7 mM MgCl_2_. A total of 20 μl sonicated liposomes was then added to the gasket, followed by mixing with a micropipette and incubation at room temperature for at least 30 min. Before use of the SLBs for measurements, 15 μl fresh HKS-M (20 mM HEPES, pH 7.4, 150 mM KCl, 1 mM MgCl_2_) was added to the gasket to account for the loss of volume due to evaporation. Unfused vesicles were washed away with HKS-M and then with reaction buffer (20 mM HEPES, pH 7.4, 100 mM KCl, 1 mM MgCl_2_).

### Mass photometry setup

All data except for the trajectories in Figs. 1a,e–g and 2e,f were acquired on a Refeyn OneMP mass photometer with a 10.8 × 2.9 μm^2^ (128 × 35 pixels) field of view. The microscope used to acquire the data in Figs. 1a,e–g and 2e,f was custom-built with a 9.4 × 6.2 μm^2^ field of view (138 × 88 pixels with a binned pixel size of 70.3 nm) and is similar to that described previously^18^. The custom-built setup is illustrated in Supplementary Fig. 15 and the differences to the setup used in our previous work are highlighted in the figure caption.

### Data acquisition

#### Dynamic mass photometry

To acquire dynamic mass photometry movies, the SLBs were placed on the sample stage to optimize the focus of the microscope. After locking the focus of the microscope, WT or ΔPRD was added to the SLB by replacing 3–6 μl buffer from the silicone gasket with 3–6 μl (total volume, 60 μl) 200 nM WT or ΔPRD (in reaction buffer) solution and mixing well with a micropipette to achieve a final concentration of 10–20 nM. Data acquisition was started ≤10 s after the addition of protein. Images were collected at 994 Hz and saved after the binning of pixels into blocks of 4 × 4 and the binning of frames into groups of 3, resulting in an effective frame rate of 331 Hz and a final pixel size of 84.4 nm (for the data used in Extended Data Figs. 2, 3, 8 and Supplementary Figs. 3 and 13 the pixel size was 77.4 nm).

Data acquisition was briefly paused (~5–10 s) once every minute (20,000 frames) to readjust the microscope focus to account for drift over time before resuming acquisition. This resulted in sets of multiple 1 min movies for each SLB and sample combination. For experiments on the effect of GTP, 3–4 movies of WT (10–20 nM) were recorded as described above, after which image acquisition was briefly paused and 1.2 μl GTP (50 mM) was added (total gasket volume, 60 μl), followed by mixing to obtain a final concentration of ~1 mM before the resumption of acquisition. For the data shown in Extended Data Fig. 8, GTP or GMPPNP was added at the beginning of the measurement together with WT (20 nM), that is, each sample condition was measured on a separate SLB. The number of replicate measurements is indicated in figure captions and corresponds to the number of sets of movies that were taken for each sample. We used the same purified batch of WT and ΔPRD for all data collected on the OneMP setup.

#### Standard mass photometry

Unless otherwise stated, standard mass photometry measurements (landing assays) were carried out in silicone gaskets (3 mm × 1 mm, GBL103250, Grace Bio-Labs) on microscopy slides that had been cleaned by consecutive sonication in Milli-Q water, isopropanol, and Milli-Q water. Protein solutions (20 μl) were added to the gaskets containing 4 μl buffer and images were acquired for 60 s at 331 Hz, except for the data in Supplementary Fig. 14c, which were acquired at 250 Hz. Landing assays were analyzed using DiscoverMP (Refeyn Ltd) to extract particle contrasts.

### Data processing

#### Background subtraction

**Extended Data Fig. 10:**
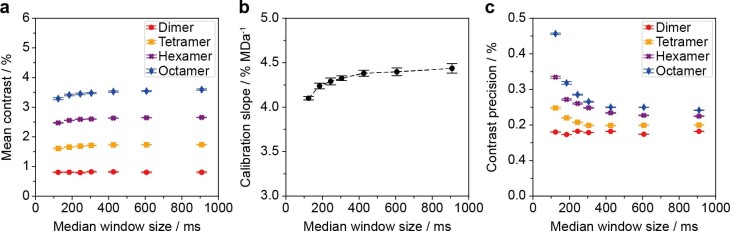
Effect of the window size of the sliding median filter on particle contrast. **(a)** Mean contrast of WT dimer (red circles), tetramer (orange squares), hexamer (purple crosses) and octamer (blue diamonds) trajectories, **(b)** mean contrast vs mass calibration slope obtained from the dynamic MP movie in (a) vs single frame length after averaging and **(c)** contrast precision of our PSF-fitting procedure for each oligomer (same symbols as in (a)) all plotted vs total exposure time of 1 frame after averaging. The plots were obtained from the same movie of WT used in Figs. 1b-d and 2a (n = 1 movie (4 min) of 20 nM WT) with additional frame averaging of 1, 2, 3, 4 and 5 frames, which corresponds to frame lengths of 3.02, 6.04, 9.05, 12.07 and 15.09 ms or frame rates of 331, 166, 110, 83 and 66 Hz, respectively (see Supplementary Information). The data in (a) and (c) are presented as mean values ± s.e.m. for each oligomeric species. The data in (b) is presented as mean values ± s.d.. For these plots n_dimer_ = 34, 51, 60, 52, 73, N_tetramer_ = 82, 102, 98, 97, 94; n_hexamer_ = 177, 229, 224, 208, 173; n_octamer_ = 22, 29, 37, 38, 33 trajectories for total exposure times of 3.0, 6.0, 9.1, 12.1, 15.1 ms, respectively. Source data

Dynamic mass photometry movies were processed by treating each frame with a sliding median background subtraction algorithm. In brief, each frame was divided by its local median, that is, the median of a pre-defined frame interval (here, 201 frames or 607 ms) centered around the frame of interest, to calculate the background-subtracted frames, *F*:

1 $$F_i = \frac{{x_i}}{{X_{i - 100:i + 100}}}$$

where *x*_*i*_ is the current raw frame and *X*_*i−*100*:i*+100_ represents the median pixel values of raw frames, from i − 100 up to (and including) i + 100. Each background-subtracted frame was then additionally treated with a two-dimensional (2D)-median noise filter to remove any large dynamic background sources (for example, fluctuations in illumination, if present). The window size of 201 frames for the sliding median algorithm was chosen because it was the smallest window size that did not detrimentally affect particle contrast or contrast precision (Extended Data Fig. 10). For smaller window sizes, particle contrast values and contrast precision decreased significantly, especially for larger particles that were less mobile, while larger window sizes increased processing times without an additional increase in sensitivity or performance. We anticipate that for slower moving particles (D < 0.3 μm^2^ s^−1^), it may be necessary to further increase the window size to avoid detrimental effects on performance. Moreover, the sliding median filter may have detrimental effects on the mass resolution at high particle densities*,* for example ≫0.4 μm^−2^ (Supplementary Fig. 3 and Supplementary Table 2). We also found that contrary to theoretical shot noise calculations, increasing the sliding median window size resulted in increasing baseline noise from 44 kDa at the minimum window size to 52 kDa at a window size of 201 frames, where it plateaued (Extended Data Fig. 9a). This trend is most likely a result of the background noise in dynamic mass photometry movies appearing as small particle-like features, which are subtracted out by the sliding median filter at small window sizes (along with particles of interest) but not at large window sizes. In practice this particle-like background noise resulted in a quantitative detection limit of ~150 kDa (Extended Data Fig. 9b,c), which prevented us from reliably quantifying dimeric particles of WT and ΔPRD. At present we have not yet identified the origin of these background features.

#### Particle detection

Particle candidates were identified by treating each processed frame with a Laplacian of Gaussian filter that matched the size of the PSFs in our mass photometry setups (Supplementary Fig. 16). From this filtered image two binary maps were constructed by applying a manually set threshold (0.0011 for all data except the data in Extended Data Figs. 2, 3, 8 and Supplementary Figs. 3 and 13, for which the threshold was set to 0.0014), and applying a local maximum filter. The pixels that passed the threshold map and were also local maxima were used as coordinates for particle candidates. For each pair of candidate coordinates, a 13 × 13 pixel region of interest was constructed with the candidate pixel at the center, and this region of interest was passed through our PSF-fitting procedure to quantify particle contrast and location. If a particle candidate was too close to an edge of the field of view to construct a 13 × 13 region of interest, that is, within 6 pixels of an edge, it was discarded. In some cases, background noise features were identified as particle candidates and this could lead to the PSF fit converging onto a nearby particle in the region of interest, which resulted in duplicate fits. To avoid problems with trajectory linking, only the first instance of a fitted particle was retained and duplicates were deleted.

#### Particle quantification and the point spread function model

The location and contrast of the particle candidates were quantified through least-squares minimization of the residual between the 13 × 13 region of interest and our PSF model (for details on how the fitting error in particle locations was extracted please refer to the Supplementary Information). Due to the interferometric nature of dynamic mass photometry, we based our PSF model on the shape of a jinc function^50^ rather than its square, which is more commonly used in fluorescence-based techniques:

2 $$I\left( r \right) = \left( {a_1{\mathrm{jinc}}\left( {\frac{r}{{w_1}}} \right) + a_2{\mathrm{jinc}}\left( {\frac{r}{{w_2}}} \right)} \right)e^{ - \left( {\frac{r}{{2\sigma }}} \right)^2}.$$

The first jinc function models the light scattered by a small particle, which is clipped by the circular objective aperture, where *r* is the distance from the PSF center, *w* the width of the jinc function and *a* its amplitude. In mass photometry setups a partial reflector positioned in the back focal plane helps to increase particle contrast by attenuating the light reflected by the coverslip^18^, which we account for by including a second jinc function. This combination of two jinc functions is then multiplied by a Gaussian with standard deviation σ, which is an empirical adjustment to reflect the appearance of the PSFs in our setups, which appear to have weaker outer lobes than we can account for with jinc functions alone. We calibrated this PSF model using standard mass photometry landing assays that were carried out ≤2 h before or after the dynamic mass photometry experiments. We then extracted and saved the ratio of the amplitudes of the two jinc functions (*a*_1_/*a*_2_), the width of the first jinc function (*w*_1_) and the standard deviation of the Gaussian (σ). The width of the second jinc function (*w*_2_) is calculated using prior knowledge of the dimensions of the back aperture and partial reflector (here, *w*_2_ = 2.27*w*_1_). The analysis of these landing assays was carried out using DiscoverMP (Refeyn Ltd), and the extracted parameters used for each measurement are supplied with the raw data.

#### Trajectory linking

The successfully fitted particles were linked into trajectories using the open-source Python package trackpy^51^. More specifically, we used the trackpy.link_df function with a maximum search distance of 4 pixels from frame to frame and a ‘memory’ of 3 frames. The memory parameter refers to the maximum number of frames during which a feature can vanish (as a result of unsuccessful PSF fitting, for example) and reappear and still be considered the same particle. Due to this memory parameter, our linked trajectories can contain gaps of up to 3 frames in length each. To obtain accurate trajectory lengths, the missing frames were treated as trajectory points at which the contrast and position could not be determined.

### Trajectory analysis

Unless otherwise stated, linked trajectories were processed as described here. Only trajectories that lasted at least 151 ms (50 frames) were used for analysis, given that this effectively reduced the amount of background noise features and incorrect linking, and improved contrast resolution (Supplementary Fig. 2), while also providing sufficient data points to calculate diffusion coefficients with high confidence. Additionally, particle trajectories that had coordinates that were within 5 pixels of the edge of the field of view were discarded to avoid artificial trajectory shortening caused by particles leaving (and sometimes re-entering) the field of view. Next, we constructed a contrast histogram for each trajectory and applied a Gaussian fit to extract the mean and standard deviation of the contrast of each trajectory. These mean trajectory contrasts were then filtered by their standard deviation to further eliminate poorly linked or noisy trajectories (Supplementary Fig. 17). For this filtering step, we used a contrast versus standard deviation trend obtained from a standard mass photometry landing assay of ΔPRD on the same instrument with eightfold frame averaging (Supplementary Fig. 17a), and applied it with an appropriate contrast offset to the trajectories obtained after length filtering (Supplementary Fig. 17b). This offset was identified by inspection to account for the additional variation caused by operating at a faster frame rate compared with standard mass photometry (offset, 0.0015 at 331 Hz). Examples of trajectories that were kept and rejected based on this filtering step are shown in Supplementary Fig. 17d,e. After these two filtering steps, the mean trajectory contrasts were plotted in histograms for WT and ΔPRD. We then used Gaussian fitting to the resulting contrast distribution to extract the mean contrast of each oligomeric species and self-calibrated the data to convert contrast to mass, and then allocated particle trajectories to the oligomeric states (that is, a trajectory was identified as belonging to a particular oligomer if its mean mass was within 2 s.d. of the mean mass of one of the oligomeric species; see Supplementary Figs. 6, 8 and 12 for examples of this selection range).

### Diffusion analysis

For each trajectory that passed the filtering steps, the cumulative probability distribution of a particle’s displacement during a lag time of 4 frames (*t* = 12 ms) was calculated (except in Fig. 2a and Supplementary Fig. 4a, where *t* = 3 ms was used to resolve multiple mobility components, if present). This lag time was chosen to reduce the influence of motion blurring, which is often referred to as a dynamic measurement error^39^, on our measurements of particle displacement. This dynamic error results in an underestimation of particle displacements at short lag times, which we observed at lag times below 12 ms (Extended Data Fig. 6). To calculate diffusion coefficients we fitted the following one-, two-, and three-component models to the calculated cumulative probability distribution, *P*(*r, t*) (ref. ^5^).

3 $$P\left( {r,\,t} \right) = 1 - e^{ - \frac{{r^2}}{{4Dt + 2\sigma ^2}}}$$

4 $$P\left( {r,\,t} \right) = 1 - we^{ - \frac{{r^2}}{{4D_1t + 2\sigma ^2}}} - \left( {1 - w} \right)e^{ - \frac{{r^2}}{{4D_2t + 2\sigma ^2}}}$$

5 $$P\left( {r,\,t} \right) = 1 - w_1e^{ - \frac{{r^2}}{{4D_1t + 2\sigma ^2}}} - w_2e^{ - \frac{{r^2}}{{4D_2t + 2\sigma ^2}}} - (1 - w_1 - w_2)e^{ - \frac{{r^2}}{{4D_3t + 2\sigma ^2}}}$$

where *r* represents particle displacement during the chosen lag time, *t*; *D*_1_, *D*_2_ and *D*_3_ represent the diffusion coefficients and *w*_1_ and *w*_2_ represent their weightings (with boundary conditions set so that the exponential weightings sum to 1); *σ* represents the 2D localization error (Fig. 1d), which is included here to avoid overestimation of particle motion at short lag times. The data in Fig. 1g and Supplementary Fig. 4a were not corrected for the localization error because corrections were found to be negligible in this case. A trajectory was characterized as having more than one mobility component if adding an additional component improved the mean squared residual of the fit by more than one order of magnitude. Using this criterion, <5% of trajectories measured in this study displayed two mobility components and none displayed three mobility components at *t* = 3 ms or 12 ms (Supplementary Figs. 4a and 5). Trajectories that satisfied at least one of the following three criteria were excluded from diffusion analysis: more than 20% of the trajectory points were gaps; the mean of all contrast values of a trajectory differed by more than 20% from the value determined by Gaussian fitting; and the trajectory was too stationary (characterized by having a fast and slow mobility component and a weighting factor of <0.3 for the fast component).

For example, in the WT dataset shown in Fig. 2a, of 343 trajectories used in the analysis, six trajectories had too many gaps (criterion 1), two trajectories had mean contrasts that differed significantly from the trajectory contrast determined by Gaussian fitting (criterion 2), and two were too stationary (criterion 3). These criteria helped eliminate trajectories that were strongly influenced by background fluctuations or were a result of incorrect trajectory linking (Supplementary Fig. 18). Using this approach, histograms of the diffusion coefficients were plotted for each oligomeric species (Supplementary Figs. 4b, 7 and 9) and the mean diffusion coefficient of each oligomer was calculated by fitting a Gaussian to these distributions. For the small number of trajectories that displayed two diffusion components, only the major component was included in these histograms. The number of histogram bins was determined using the Freedman–Diaconis rule.

### Residence time analysis

To calculate the dissociation rate constants of each oligomeric species from the SLB, we slightly modified the trajectory filtering procedure described above (Supplementary Information). After filtering the detected trajectories, contrast histograms (70 bins) were plotted and trajectories were sorted by oligomeric species as described above. The distribution of trajectory lengths of a given oligomeric species was fitted to the probability density function of a 1-component exponential distribution and the rate parameter was optimized by maximum likelihood estimation. To correct for the threshold of 33 ms (10 frames) that was applied in prior filtering steps, it was necessary to scale the probability density function by incorporating the threshold as an additional parameter:

6 $$p(k|t,t_d) = ke^{ - k\left( {t - t_d} \right)}$$

where *k* is the dissociation rate constant from the membrane, *t*_*d*_ is the time threshold applied during filtering, and *t* is the trajectory length. This process was repeated for each oligomeric species detected in the dynamic mass photometry measurements of WT (7 repeats) and ΔPRD (4 repeats) taken at 10–20 nM (Supplementary Figs. 10 and 11).

### GTP data analysis

For details on how the data examining the effect of GTP and GMPPNP on the oligomeric distribution of WT were analyzed (for example, modified trajectory filtering), please refer to the Supplementary Information.

### Simulations of dynamic mass photometry movies

For details on how simulations of dynamic mass photometry movies were carried out, please consult the Supplementary Information.

### Reporting Summary

Further information on research design is available in the Nature Research Reporting Summary linked to this article.

## Online content

Any methods, additional references, Nature Research reporting summaries, source data, extended data, supplementary information, acknowledgements, peer review information; details of author contributions and competing interests; and statements of data and code availability are available at 10.1038/s41592-021-01261-w.

## Supplementary information

Supplementary Information Supplementary Methods, Supplementary Figs. 1–18, Supplementary Tables 1–5

Reporting Summary

Supplementary Video 1 15 s section of the dynamic mass photometry movie (displayed at 25 frames per second) of WT (20 nM) in contact with an SLB, corresponding to the data shown in Figs. 1b,c and 2a. Red dots represent the locations of signals that were successfully identified as particles and quantified. Scale bar, 1 μm.

Supplementary Video 2 Section (frames 3797–9974) of a dynamic mass photometry movie of WT in contact with an SLB, in which the particle trajectory shown in Fig. 1e–g was identified (circled in red). This particle frequently approaches the edge of the field of view, which intermittently prevented detection and quantification by our processing software (indicated by the red circle briefly disappearing), and resulted in its trajectory being split up into several shorter ones. As such, the short sub-trajectories had to be combined into the particle’s entire trajectory by inspection. In this experiment, 6 μl 1 μM WT was added to 60 μl buffer on the SLB but with minimal mixing, which led to a gradual increase in particle density over time. The movie is displayed at 25 frames per second. Scale bar, 1 μm.

Supplementary Video 3 Zoom-in on a later section (frames 15900–16300) of the dynamic mass photometry movie shown in Supplementary Video 2, showing the dissociation event displayed in Fig. 2e. The movie is displayed at 25 frames per second. Scale bar, 1 μm.

Supplementary Video 4 Zoom-in on a section (frames 22500–22800) of a dynamic mass photometry movie of ΔPRD (10 nM) in contact with an SLB, showing the association event displayed in Fig. 2f. The movie is displayed at 25 frames per second. Scale bar, 1 μm.

Supplementary Data 1 Source data for Supplementary Figures

## Data Availability

The raw and processed data have been deposited in the University of Oxford Research Archive (10.5287/bodleian:Qm2vgeo5z). This dataset contains all raw movies that were used in this study, along with the corresponding background-subtracted dynamic mass photometry movies. Additionally, it contains spreadsheets with the data of all particles that were successfully detected, quantified, and linked into trajectories by our software for each movie. Details on the raw data, processed data and data points used for each Figure and Supplementary Figure and the mass photometry landing assays used to calibrate the point spread function model for dynamic mass photometry (along with the calibration settings) can be found in the summary spreadsheet. All Figures except Fig. 1a and Supplementary Figs. 1, 15 and 16 have associated raw data. Source data are provided with this paper.

## Source data

Source Data Fig. 1 Statistical Source Data for Fig. 1
Source Data Fig. 2 Statistical Source Data for Fig.2
Source Data Extended Data Fig. 2 Statistical Source Data for Extended Fig. 2
Source Data Extended Data Fig. 3 Statistical Source Data for Extended Fig. 3
Source Data Extended Data Fig. 4 Statistical Source Data for Extended Fig. 4
Source Data Extended Data Fig. 5 Statistical Source Data for Extended Fig. 5
Source Data Extended Data Fig. 6 Statistical Source Data for Extended Fig. 6
Source Data Extended Data Fig. 7 Statistical Source Data for Extended Fig. 7
Source Data Extended Data Fig. 8 Statistical Source Data for Extended Fig. 8
Source Data Extended Data Fig. 9 Statistical Source Data for Extended Fig. 9
Source Data Extended Data Fig. 10 Statistical Source Data for Extended Fig. 10

## Extended data

are available for this paper at 10.1038/s41592-021-01261-w.

## Code availability

The Python software used for image processing, and for particle identification, fitting and trajectory linking is available on request, along with the jupyter notebooks that outline the use of this package, from raw movies to linked particle trajectories, as well as the manual settings used for every dataset. We can also provide the jupyter notebooks that were used to extract mass distributions, diffusion coefficients and dissociation rate constants from the spreadsheet of linked trajectories, which includes all filtering steps as described in the Methods section. From these notebooks, all quantitative Figures and Supplementary Figures in this paper can be generated.
