# Supplementary material for: Mass photometry enables label-free tracking and mass measurement of single proteins on lipid bilayers
Source: Nat Methods. 2021 Oct 4;18(10):1247–52. doi: 10.1038/s41592-021-01261-w (PMC8490153; doi:10.1038/s41592-021-01261-w)
Supplement: Supplementary file 1 — Supplementary Methods, Supplementary Figs. 1–18, Supplementary Tables 1–5 [file 41592_2021_1261_MOESM1_ESM.pdf]

nature portfolio

https://doi.org/10.1038/s41592-021-01261-w

Supplementary information

Mass photometry enables label-free tracking and mass measurement of single proteins on lipid bilayers

In the format provided by the authors and unedited

Supplementary Information

Mass photometry enables label-free tracking and mass measurement of single proteins on lipid bilayers

Eric D. B. Foley[#†], Manish S. Kushwah[#†], Gavin Young[†‡] and Philipp Kukura[†*]

[#]Equal contribution

[†]Physical and Theoretical Chemistry Laboratory, Department of Chemistry, University of Oxford, UK

[‡]Current address: Refeyn Ltd., Oxford, Oxfordshire, GB

*To whom correspondence should be addressed: philipp.kukura@chem.ox.ac.uk

Contents

Materials 1

Buffer preparation 1

Protein expression and purification 2

Size-exclusion chromatography and mass photometry measurements. 3

Additional frame averaging and PSF fitting error of particle locations 3

Trajectory filtering for residence time and GTP analysis. 4

Simulation of dynamic MP movies. 5

Supplementary Figure 1 6

Effect of background subtraction on baseline noise

Supplementary Figure 2 7

Effect of filtering particle trajectories by their SLB residence times

Supplementary Figure 3 8

Simulated effect of motion blur and particle density on particle contrast in dynamic MP

Supplementary Figure 4 9

Extraction of diffusion coefficients from trajectories

Supplementary Figure 5 10

Distribution of mobility components

Supplementary Figure 6 11

Mass distribution of WT

Supplementary Figure 7 12

Distributions of diffusion coefficients of WT oligomers

Supplementary Figure 8 13

Mass distribution of ∆PRD

Supplementary Figure 9 14

Distribution of diffusion coefficients of ∆PRD oligomers

Supplementary Figure 10 15

Distribution of trajectory residence times of ∆PRD oligomers on the SLB

Supplementary Figure 11 16

Distribution of trajectory residence times of WT oligomers on the SLB

Supplementary Figure 12 17

Effect of GTP addition on the mass distribution of WT

Supplementary Figure 13 18

Effect of ultracentrifugation on the measured oligomeric distribution of WT

Supplementary Figure 14 19

Effect of size exclusion chromatography on the abundance of dynamin oligomers.

Supplementary Figure 15 20

Custom-built setup used in this study

Supplementary Figure 16 21

Image processing for identification of particle candidates

Supplementary Figure 17 22

Filtering of trajectories using the standard deviation of their contrast distributions

Supplementary Figure 18 23

Examples of particles that were excluded from the diffusion analysis

Supplementary Tables 24

Supplementary Table 1.

Supplementary Table 2.

Supplementary Table 4.

Supplementary Table 5.

References 25

Materials

HEPES (H3375), KCl(P9541), Imidazole (I5513), DL-Dithiothreitol (DTT: D9779), Magnesium Chloride hexahydrate (M2670), Glycerol (G5516), Isopropyl-β-D-thiogalactoside (IPTG: I6758), Ampicillin (59349), Chloramphenicol (C0378), Protease inhibitor cocktail (4693159001; Roche), HiTrap®TALON® cobalt columns (GE28-9537-67), StrepTrap™ columns (GE28-9075-48), Grace Bio-Labs reusable CultureWell™ silicone gaskets (GBL103280), Chloroform (288306) and Desthiobiotin (71610-3) were purchased from Merck Life Science UK Limited. Glass coverslips (24x50 mm, Menzel Gläser, VWR 630-2603) were purchased from Thermo Fisher Scientific. 1,2-dioleoyl-sn-glycero-3-phosphocholine (DOPC; 850375P) and 1,2-dioleoyl-sn-glycero-3-phospho-L-serine (sodium salt) (DOPS; 840035P) were purchased from Avanti Polar Lipids. GTP (NU-1012) and GppNHp - Tetralithium salt (GMPPNP) (NU-401) were purchased from Jena Biosciences. BL21-CodonPlus and Terrific Broth were purchased from Agilent Technologies and Fisher Scientific, respectively. The ultrasonicator and plasma cleaner are from Sonics & Materials, Inc. and Diener electronic, respectively. The Dynamin1 wild type (WT) bacterial expression vector[1,2] was generously provided by Prof. Thomas Pucadyil (IISER Pune, India). ΔPRD ($Dyn1_{1\text{-}746}$) was generated in the lab using standard cloning procedures and confirmed using DNA sequencing.

HEPES, KCl, $MgCl_2$, and Imidazole, stocks were prepared in Milli-Q® water (18.2 MΩ·cm) (Milli-Q) and filtered using a 0.2 μm filters. Ampicillin, IPTG and DTT were prepared in degassed Milli-Q, aliquoted and stored at -20°C. GMPPNP (50 mM) was dissolved in degassed 20 mM HEPES (pH = 7.4). GTP solution and GMPPNP stocks were aliquoted and stored at -20°C. DOPC (25 mM) and DOPS (10 mM) stocks were prepared using chloroform and aliquoted and stored at -20°C under nitrogen.

Buffer preparation

Due to its label-free nature, dynamic mass photometry (MP) is sensitive to impurities originating from stock solutions used to prepare buffers, which can integrate into the SLBs and cause defects or additional background noise. Therefore, buffer stocks were prepared carefully. HEPES (1 M, pH = 7.7), KCl (3 M) and $MgCl_2$ (1 M) stocks were prepared in degassed Milli-Q water and filtered twice (0.2 μm, VWR international, 514-600) and stored at 4°C until use. Buffers were reconstituted form the stock solutions immediately before dynamic MP experiments with degassed Milli-Q water, and replaced every 2-3 hours to decrease the influence of reactive oxygen species during dynamic MP measurements.

Protein expression and purification

Bacterial expression vectors for WT and ΔPRD were transformed in BL21-CodonPlus and grown until OD_{600} = 0.6 in Terrific Broth containing ampicillin (100 μg ml^{-1}) at 37°C. Protein production was induced with 0.1 mM IPTG, and bacterial cultures were grown for 12 hours at 18°C. Cells were harvested, and bacterial pellets were stored at -80°C. For purification, bacterial pellets were thawed on ice and dissolved in lysis buffer (20 mM HEPES, pH = 7.4, 300 mM KCl and 20 mM Imidazole) containing one tablet of protease inhibitor cocktail. Cells were lysed using a cell homogenizer, and lysate was spun at 20,000 g for 20 min at 4°C to remove insoluble cell debris. The supernatant was applied to a 5 ml HiTrap®TALON® column. The column was washed with 60 ml lysis buffer, followed by 60 ml wash buffer (20 mM HEPES, pH = 7.4, 150 mM KCl and 20 mM Imidazole). The protein was eluted with elution buffer (20 mM HEPES, pH = 8, 150 mM KCl, 200 mM Imidazole) and eluted protein was applied to a 5 ml StrepTrap™ column, which was pre-equilibrated with elution buffer, at 2 ml min^{-1}. The column was washed sequentially with 50 ml strep-1 buffer (20 mM HEPES, pH = 7.4, 300 mM KCl), 50 ml strep-2 buffer (20 mM HEPES, pH = 7.4, 300 mM KCl, 1 mM DTT) and 50 ml strep-3 buffer (20 mM HEPES, pH = 7.4, 150 mM KCl, 1 mM DTT). The protein was eluted with strep-elution buffer (20 mM HEPES, pH = 7.4, 150 mM KCl, 1mm DTT, 10% glycerol, 2.5 mM Desthiobiotin), flash frozen and stored at -80°C until use.

To prepare the protein for dynamic MP experiments, WT or ΔPRD were dialysed overnight at 4°C in degassed HKS-150 buffer (20 mM HEPES, pH = 7.4, 150 mM KCl) containing 1 mM DTT. After dialysis, the protein was spun at 20,000 g for 30 minutes at 4°C to remove aggregates. The supernatant was collected, and the concentration was estimated by measuring absorbance at OD_{280} nm (ε_{280} = 62,800 M^{-1} cm^{-1} and 54,300 M^{-1} cm^{-1} for WT and ΔPRD, respectively). Proteins were stored on ice during use. We determined that the protein stocks contained no misfolded or aggregated proteins by performing standard MP and dynamic MP measurements on protein samples that had been centrifuged at 20,000 g and 100,000 g (Supplementary Figure 13). Additionally, we purified our ΔPRD stock by size exclusion chromatography and again found no evidence of aggregation (Supplementary Fig. 14). Finally, the increase in membrane contact, with simultaneous decrease in diffusion coefficient and increase in membrane affinity as a function of increasing dynamin oligomerisation (Fig. 2b, d) also suggests that oligomers observed in our study are fully-folded, membrane-active oligomers.

Size-exclusion chromatography and mass photometry measurements.

Dynamin1-ΔPRD (13 µM; 1 ml) was thawed from -80°C (stored in 20 mM HEPES, pH = 7.4, 150 mM KCl, 10% glycerol, 1 mM DTT and 2.5 mM desthiobiotin) and applied to a Superdex 16/600 200 column which was equilibrated with degassed HKS-150 (20 mM HEPES, pH = 7.4, 150 mM KCl). 1 ml fractions were collected at 1 ml/min and 1 mM DTT was added to all fractions. The concentration of each fraction was measured using absorbance at OD_{280} nm and 20 µl from each fraction was assessed by SDS-PAGE. All fractions were stored on ice until MP measurements. For standard MP measurements, each fraction was either diluted to 200 nM using HKS-150 supplemented with 1 mM $MgCl_2$ and 1 mM DTT or measured without any dilution (low protein concentration elutions). Diluted samples, were incubated for one minute at RT prior to measurement and for undiluted samples 200 µl of the elution was incubated at RT for 5 minutes and repeat measurements were taken from the 200 µl stock.

Additional frame averaging and PSF fitting error of particle locations

For every successful PSF-fitting operation, the error of the fit in x and y was estimated from the covariance of the parameters of the fit. To determine the mean fitting errors in 2 dimensions for each oligomeric species of WT, we examined the data shown in Fig. 1b-c at different effective imaging speeds by averaging together increasing numbers of consecutive frames (2, 3, 4 and 5, resulting in effective frame rates of 166 Hz, 100 Hz, 83 Hz and 66 Hz) of the dynamic MP movie after treatment with the sliding median filter. Trajectories were linked and filtered as described in the Methods section (with incrementally increasing offsets from 0.0015-0.0023 for frame rates from 331 Hz to 66 Hz). The median PSF fitting error in x and y was then determined for each trajectory that passed the filtering steps and a Gaussian was fit to the resulting distribution of median x and y errors of each oligomeric species to extract the mean localisation error and standard deviation. The fitting errors in 2D particle positions displayed in Fig. 1d were calculated from the means of these Gaussian fits. We note that this provides a lower bound on the localisation precision of dynamic MP at our operating frame rate. Trajectory linking parameters were adjusted for decreased effective frame rates (max search distance of 4, 6, 6, 7 and 8, and memory of 3, 2, 2, 1 and 1 for 331 Hz, 166 Hz, 100 Hz, 83 Hz and 66Hz, respectively).

Trajectory filtering for residence time and GTP analysis

Residence time analysis

To calculate the dissociation rate constants from the SLB of each oligomeric species, we slightly modified the trajectory filtering procedure described in the Methods section to limit the impact of background noise on measured trajectory lengths. First, we constructed spatial maps of the probability density of detected particle locations (scipy.stats.gaussian_kde) across the field of view for each movie to identify regions that showed anomalously high particle densities (probability density ≥ 0.008, often a sign of improperly bound or immobile particles). Trajectories that contained less than ten points (>33 ms in length) outside of these identified regions were discarded. Due to the reduced number of trajectory points in this analysis (≥ 10 points instead of ≥ 50 points), we determined the contrast of each trajectory by taking the median of all its contrast measurements instead of Gaussian fitting as described in the Methods section. Trajectories were then filtered using the interquartile range of their contrast in a similar way as described above (offset = 0.0025, Supplementary Fig. 17a-b). Additionally, trajectories that had coordinates within 5 pixels of the edge of the field of view or contained any gaps were discarded to ensure that trajectory lengths were as accurate as possible.

Analysis of the effect of GTP and GMPPNP on the oligomeric distribution of dynamin

For the analysis of the effect of GTP on the mass distribution of WT on the SLB (Fig. 2g, Extended Data Fig. 8) the trajectories were filtered as described in the residence time analysis section, except that a minimum trajectory length threshold of 20 instead of 10 frames was applied to limit the effect of transient background fluctuations on the count of dimeric particles. Additionally, trajectories containing gaps were not discarded. Mass histograms were plotted for the movie recorded immediately before GTP was added (last 1 min of acquisition before GTP addition) and the first movie recorded immediately after GTP addition (first minute of acquisition after addition). Trajectories were allocated to a particular oligomeric species if their mass, was within two standard deviations of the mean mass of that oligomer, which was determined from the entire set of 1 min movies up to GTP addition in each case by Gaussian fitting (Supplementary Fig. 6b-f). This procedure was used for five replicate measurements of WT-dynamin (10-20 nM to achieve satisfactory particle density) with GTP addition (1 mM) after 3-4 min of acquisition (Supplementary Fig. 12). We used the same approach for the data shown in Extended Data Fig. 8, except that GTP or GMPPNP were added together with WT at the beginning of the measurement rather than after a few minutes of data acquisition

Simulation of dynamic MP movies

The graphs in Supplementary Fig. 3 and Extended Data Fig. 3 were obtained from a set of simulated dynamic MP movies, which were generated by simulating PSFs onto raw images of HKS-100 buffer in contact with an SLB (20,000 frames recorded at 331 Hz for) and then processing the resulting images with the sliding median filter (window size = 201 frames, 607 ms). To simulate PSFs, we used the model described in Equation 2 (a_1/a_2 = -5.4315762370, w_1 = *2.1800050329*, σ = 5.0012142342). This PSF model was scaled appropriately to obtain contrasts representative of WT dimer, tetramer, hexamer and octamer (1.0, 2.0, 3.0 and 4.0%, respectively). Diffusion and motion blur were simulated by moving the PSFs incrementally in x and y every 0.2 ms (or exactly between every frame for simulations without motion blur) by sampling x and y step sizes from a Gaussian distribution centred around 0 and with a standard deviation of 2Dt, where D is the diffusion coefficient of the corresponding WT species (Extended Data Fig. 5, Supplementary Table 4) and t the lag time (0.2 ms for motion blur and 3.0 ms in the absence of motion blur). Trajectory lengths were determined by sampling from an exponential distribution with a rate constant of 20 s^{-1} and each oligomeric species was assigned an equal probability of appearing on the SLB. The resulting movies were analysed as described in the residence time analysis section but without spatial filtering for high particle density regions.

Supplementary Figure 1

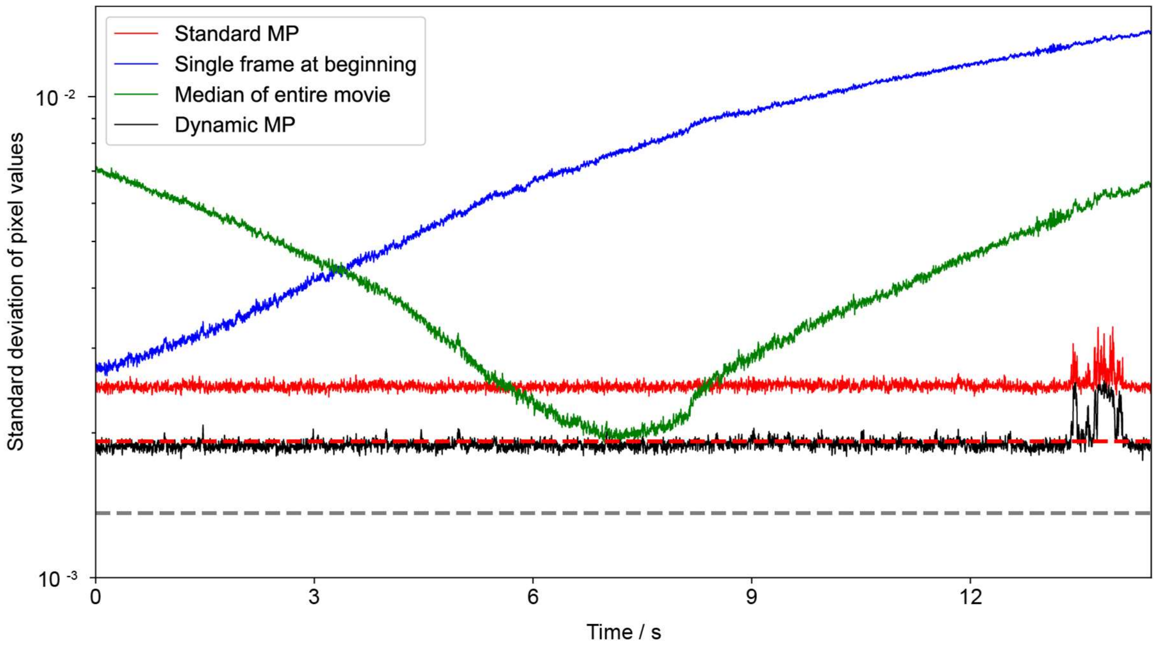

Effect of background subtraction on baseline noise

The baseline noise (the standard deviation of all pixel values in a given frame) of a dynamic MP movie of buffer solution on an SLB obtained with the background subtraction used in standard MP (red line), by subtracting the initial frame from all frames (blue line), subtracting the median of the entire movie from all frames (green line) and using the sliding median filter used for the dynamic MP experiments in this work (black line). The grey dashed line shows the theoretical shot noise limit when using the sliding median filter. The theoretical shot noise limit when using standard MP background subtraction (red dashed line) overlaps with the sliding median baseline noise (~0.002). The sliding median background subtraction results in lower background noise because the background is calculated from 200 additional frames compared to the background subtraction used in standard MP. Occasional spikes in background noise appear due to larger particles diffusing across the field of view.

Supplementary Figure 2

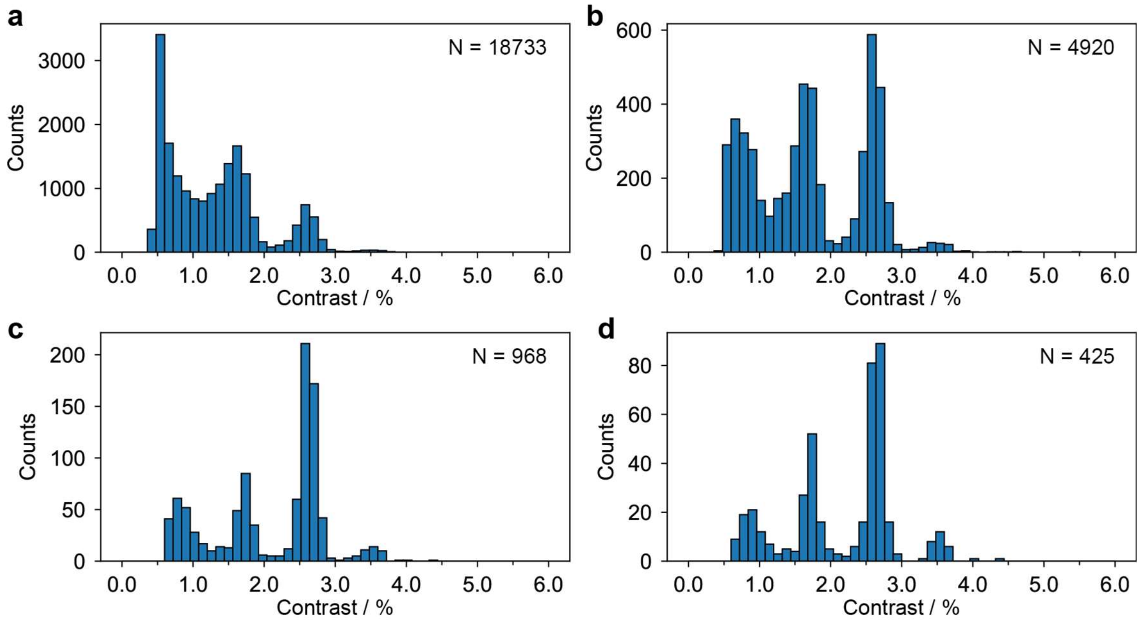

Effect of filtering particle trajectories by their SLB residence times

(a)-(d) Contrast histograms (bin width = 0.12%) obtained from the same dynamic MP movie of WT used in Fig. 1b-d and 2a after applying a minimum threshold of 5, 10, 30 and 50 frames (331 Hz) to the trajectory length, respectively. Particle trajectories that remained on the membrane for fewer frames than the specified threshold were discarded. Here, each data point corresponds to the median contrast of a trajectory. This length filtering procedure effectively improves the quality of the data and reduces background features that were incorrectly identified as particles and linked into short trajectories, resulting in an increase in contrast resolution.

Supplementary Figure 3

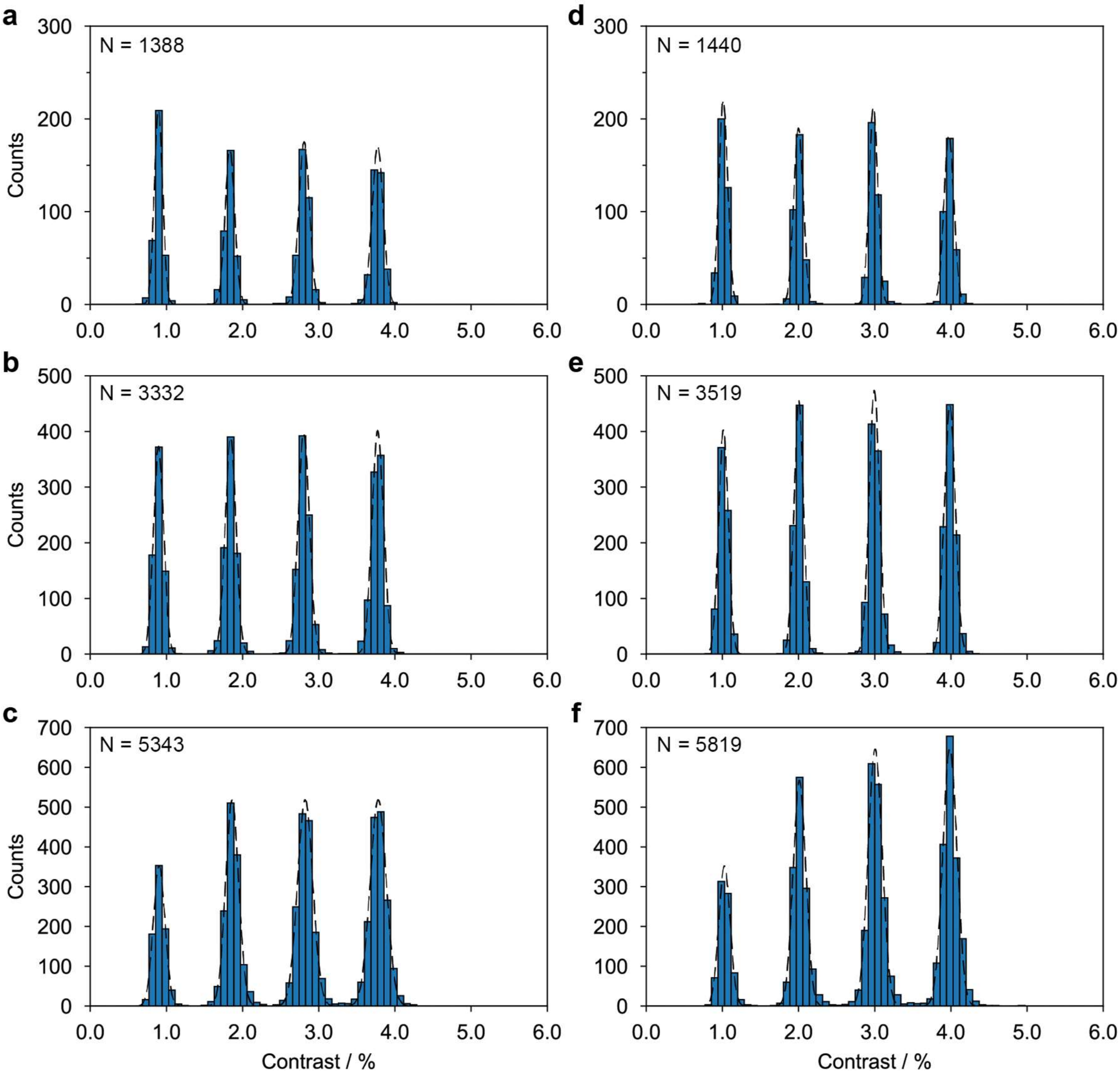

Simulated effect of motion blur and particle density on particle contrast in dynamic MP

(a)-(c) Contrast histograms generated from a movie of HKS-100 buffer in contact with a supported lipid bilayer (331 Hz) with point spread functions with contrasts of 1.0-4.0% roughly corresponding to WT dimer, tetramer, hexamer and octamer simulated with motion blur onto the raw images with mean particle densities of 0.13, 0.30 and 0.79 μm^{-2}, respectively. Motion blur was simulated by allowing the PSFs to move every 0.2 ms (~15 times in each image) based on the diffusion components measured for each species in dynamic MP experiments and each oligomeric species was assigned the same probability of appearing in the movie and the same membrane affinity (see methods) **(d)-(f)** Contrast histograms of dynamic MP movies with simulated PSFs generated in the same way as in (a)-(c) but without motion blur, *i.e.* particle movement occurred exactly in between acquired images. We note that at the highest particle density (c, f) there is a bias in the detection algorithm towards larger species and peak widths are larger than at lower particle densities. The increase in peak widths may be a consequence of the sliding median filter or decreased precision in particle fitting due to an increased amount of overlapping PSFs or simply due to less precise PSF fitting due to more crowded images. The dynamic MP experiments in this study exhibited particle densities around 0.3-0.5 μm^{-2}, *i.e.* similar to those in (b) and (e). Only trajectories that lasted at least 10 frames were used to generate this figure and the mean contrast and standard deviation for each species in these histograms are supplied in Supplementary Table 2. Black dashed lines represent Gaussian fits to the contrast histograms.

Supplementary Figure 4

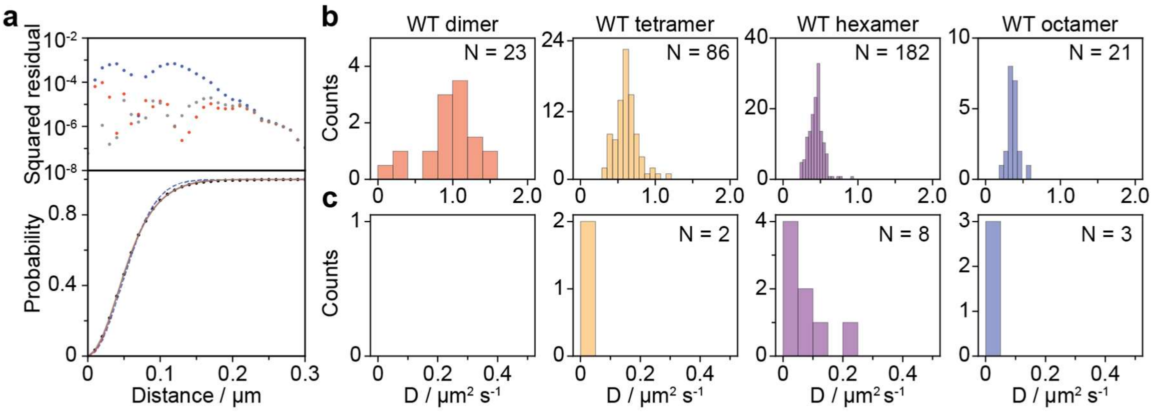

Extraction of diffusion coefficients from trajectories

(a) 1-(blue), 2-(red) and 3-(gray) component fits to the cumulative probability density of the distance moved during one frame (3 ms) by the particle shown in Fig. 1e-g (N = 6033 measurements of 1-frame displacements). **(b)** Distributions of the major diffusion component of WT dimer (red), tetramer (orange), hexamer (purple) and octamer (blue) particles for the same data used in Fig. 1b-d and 2a (n = 1, 4 min movie of the same sample). **(c)** Same as **(b)** for the minor diffusion component (if present). The vast majority of particles only exhibited one diffusion component (Supplementary Fig. 5).

Supplementary Figure 5

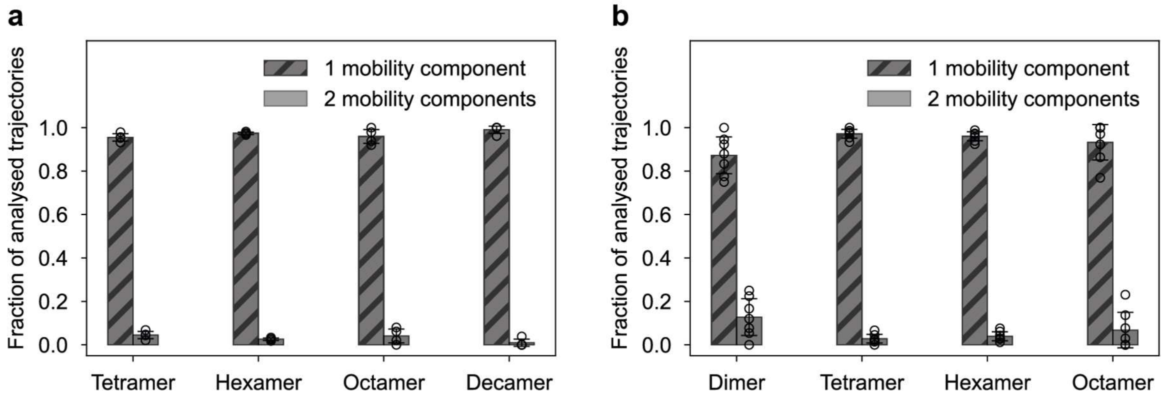

Distribution of mobility components

Mean fractions of one- and two-component diffusion of each oligomeric species of ΔPRD (n = 4 independent measurements with a total of $N_{tetramer}$ = 214, $N_{hexamer}$ = 937, $N_{octamer}$ = 330, $N_{decamer}$ = 83) **(a)** and of WT (n = 7 independent measurements with a total of N_{dimer} = 197, $N_{tetramer}$ = 501, $N_{hexamer}$ = 1326, $N_{octamer}$ = 156) **(b)** based on fits to the cumulative probability density function of particle displacement with a lag time of 12 ms (see methods). Only trajectories that lasted at least 50 frames were considered for this plot. The contrast of WT dimer overlaps with the contrast of background features, which is most likely why it shows increased two-component diffusion compared to the other species. Due to its lower contrast, the ΔPRD dimer could not be detected and is thus not included in this plot. Error bars are presented as mean values ± standard deviation.

Supplementary Figure 6

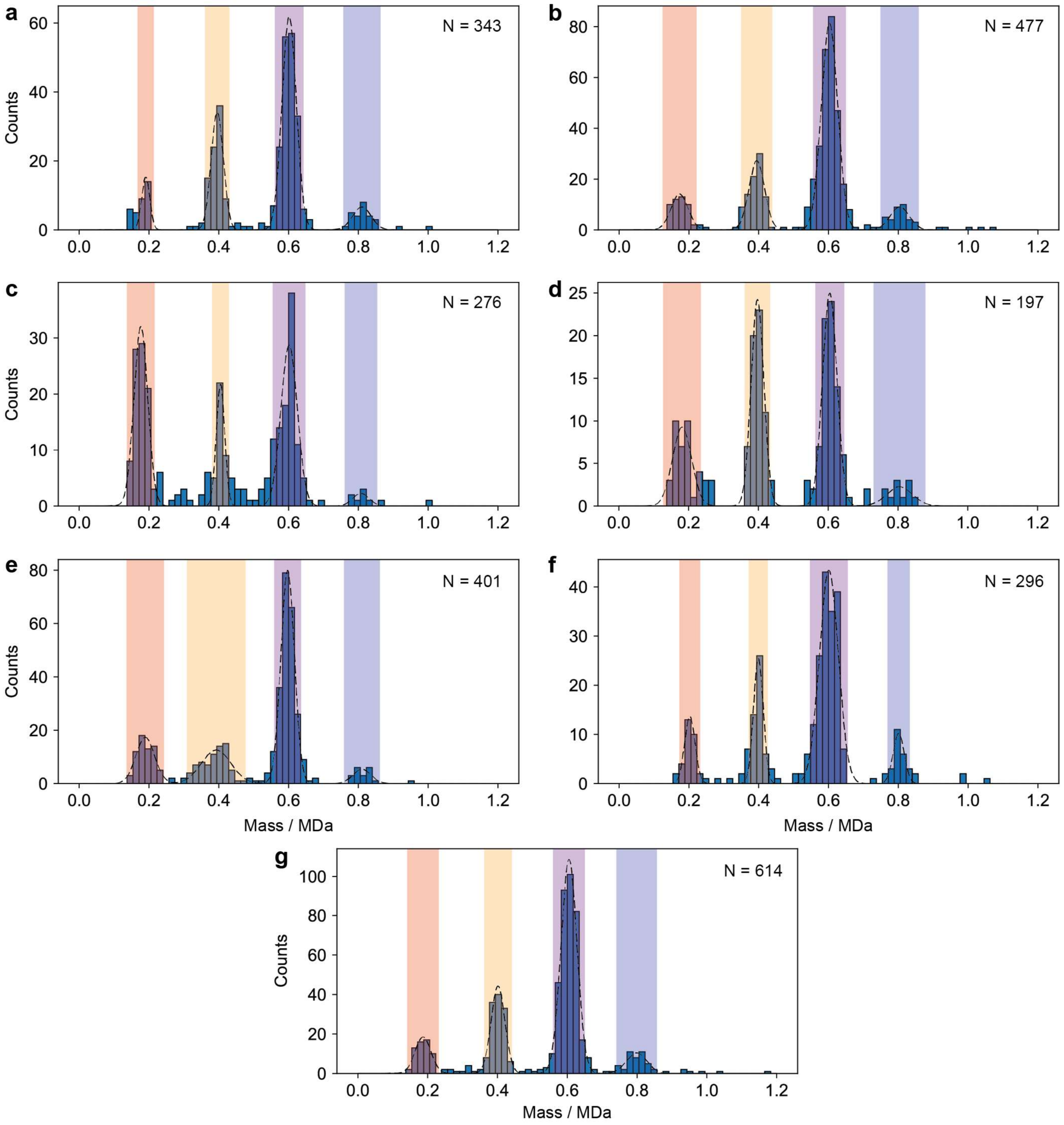

Mass distribution of WT

(a-g) Mass histograms obtained from seven repeats of dynamic MP measurements (4-5 min each) of WT (10-20 nM) in contact with an SLB. Each data point in the histograms represents the mean contrast of a trajectory as determined by Gaussian fitting, as described in the methods section. The black dashed lines represent Gaussian fits to the oligomeric peaks of WT. If a trajectory's mean contrast was within two standard deviations of the mean of these Gaussian fits, it was classified as that particular oligomeric species (red = dimer, orange = tetramer, purple = hexamer, blue = octamer). The number of bin width for all mass histograms was set to 0.017 MDa (70 bins) and the same initial guess for Gaussian fitting was used in each case. The dimer trajectories often overlapped in mass with background features (see also Extended Data Fig. 10b-c) and were thus difficult to distinguish from background noise.

Supplementary Figure 7

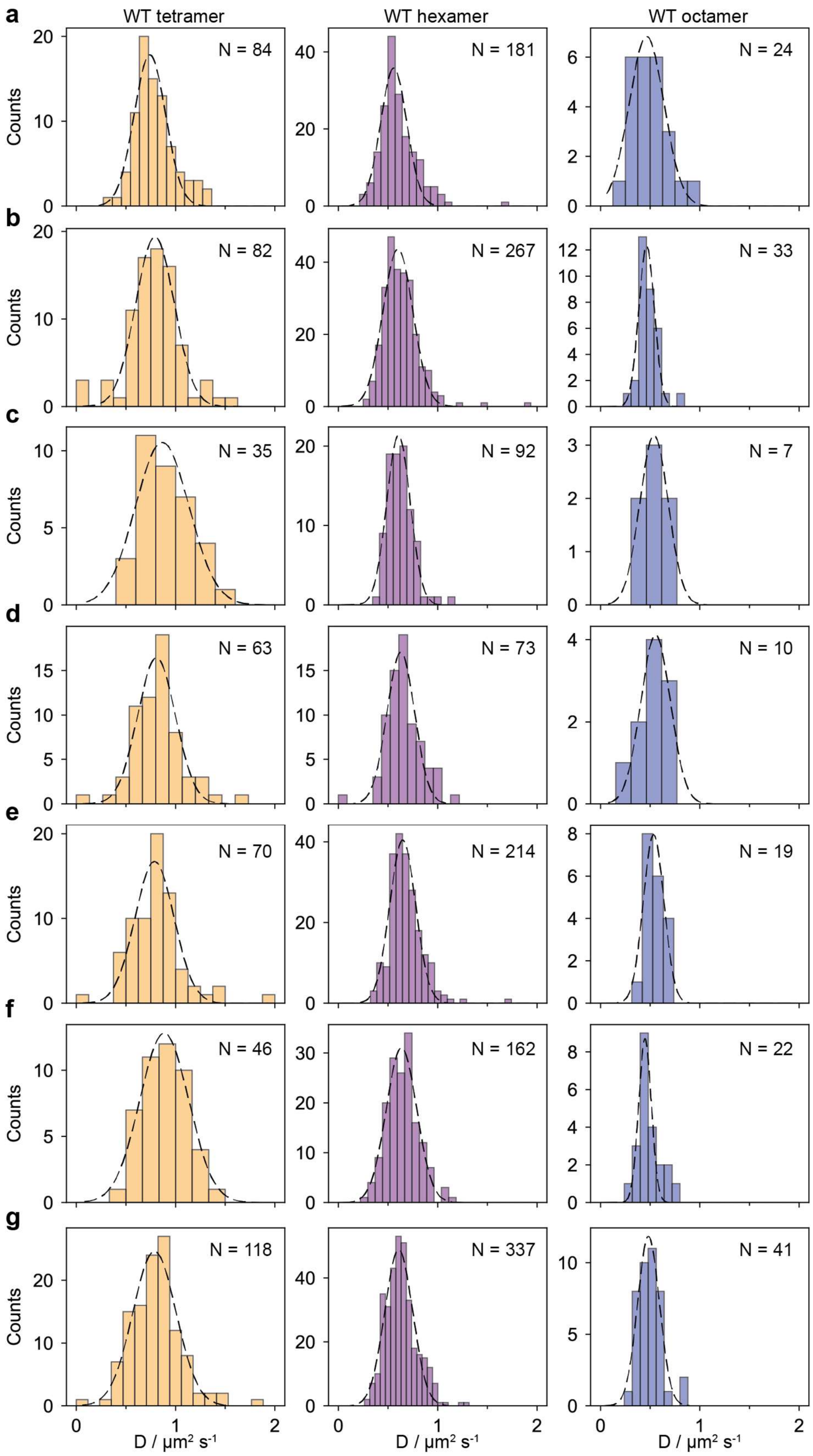

Distributions of diffusion coefficients of WT oligomers

(a-g) Diffusion coefficients calculated as described in the methods section for each oligomeric species (tetramer = orange, hexamer = purple, octamer = blue) detected in the WT measurements shown in Supplementary Fig. 6. The mean diffusion coefficients were determined by Gaussian fitting to these histograms (black dashed line). The number of bins in each histogram was chosen using the Freedman-Diaconis rule.

Supplementary Figure 8

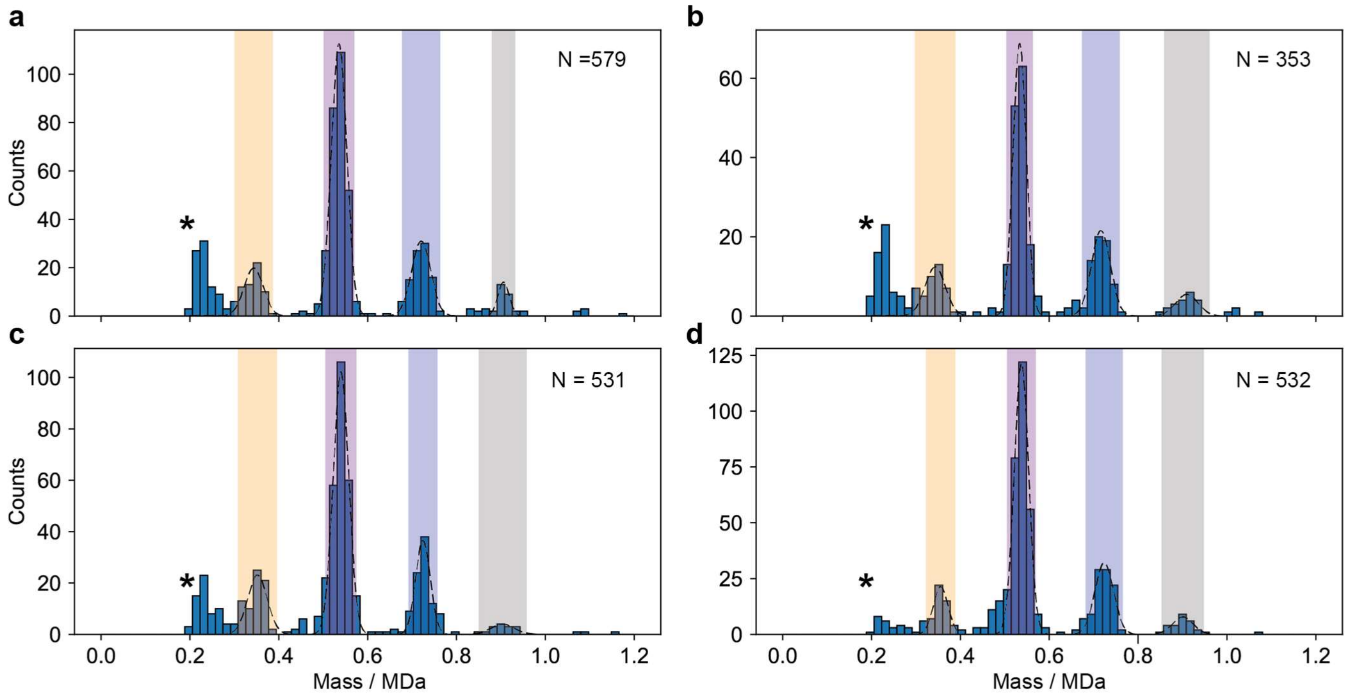

Mass distribution of ∆PRD

(a-d) Mass histograms obtained from four repeats of dynamic MP measurements (4-5 min each) of ∆PRD (10-20 nM) in contact with an SLB. Each data point in the histograms represents the mean contrast of a trajectory as determined by Gaussian fitting, as described in the methods section. The black dashed lines represent Gaussian fits to the oligomeric peaks of ∆PRD. If a trajectory's mean contrast fell within two standard deviations of the mean of these Gaussian fits, it was classified as that particular oligomeric species (orange = tetramer, purple = hexamer, blue = octamer, grey = decamer). The bin width in all histograms was set to 0.016 MDa and the same initial guess for Gaussian fitting was used in each case. *Detected events due to background noise representing the limit of detection in these measurements. As such, the ∆PRD dimer (180 kDa) could not be reliably detected.

Supplementary Figure 9

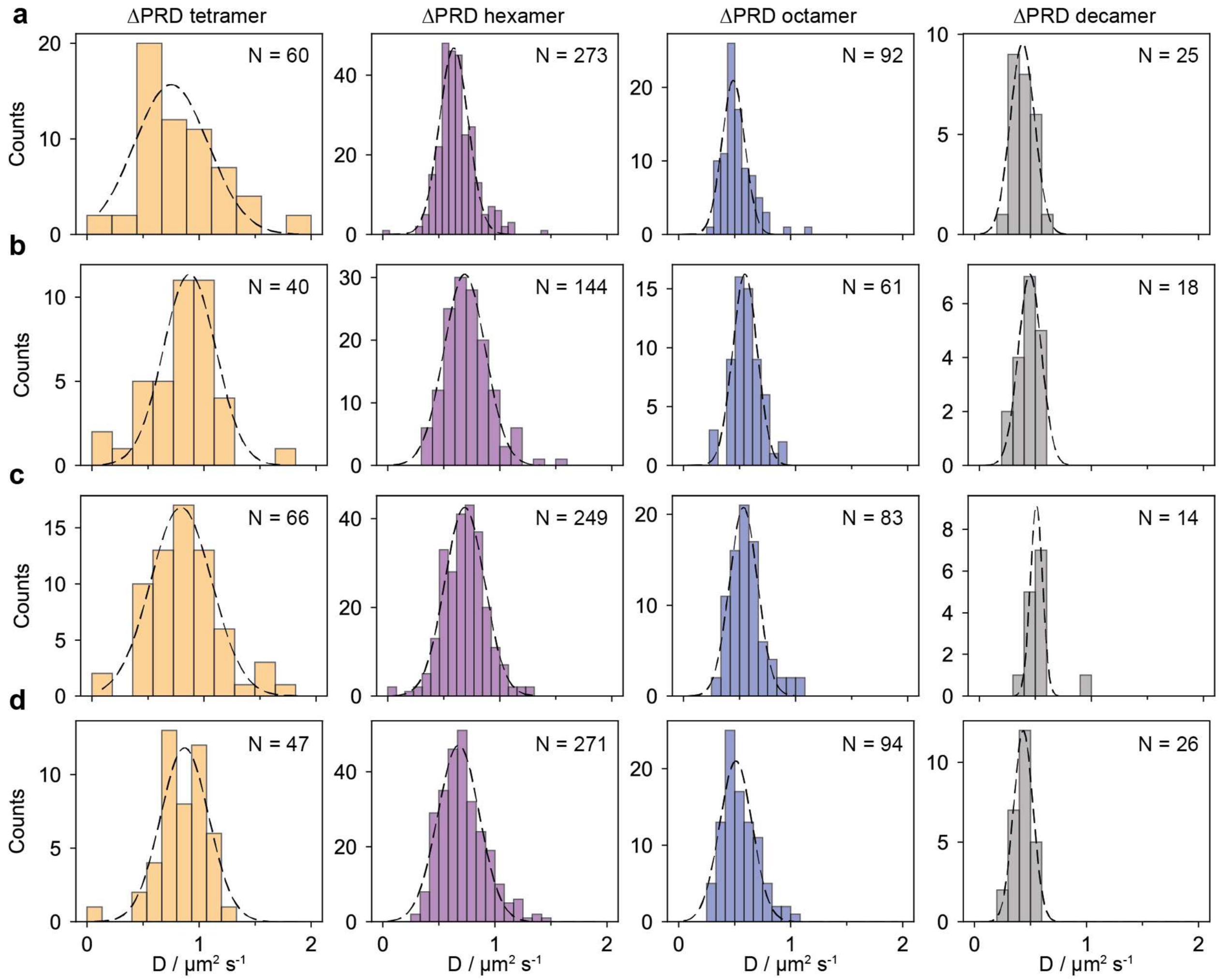

Distribution of diffusion coefficients of ΔPRD oligomers

(a-d) Diffusion coefficients calculated as described in the methods section for each oligomeric species (tetramer = orange, hexamer = purple, octamer = blue, decamer = gray) of the ΔPRD measurements shown in Supplementary Fig. 8. The mean diffusion coefficients were determined by Gaussian fitting (black dashed line) to the histograms. The number of bins in each histogram was chosen using the Freedman-Diaconis rule.

Supplementary Figure 10

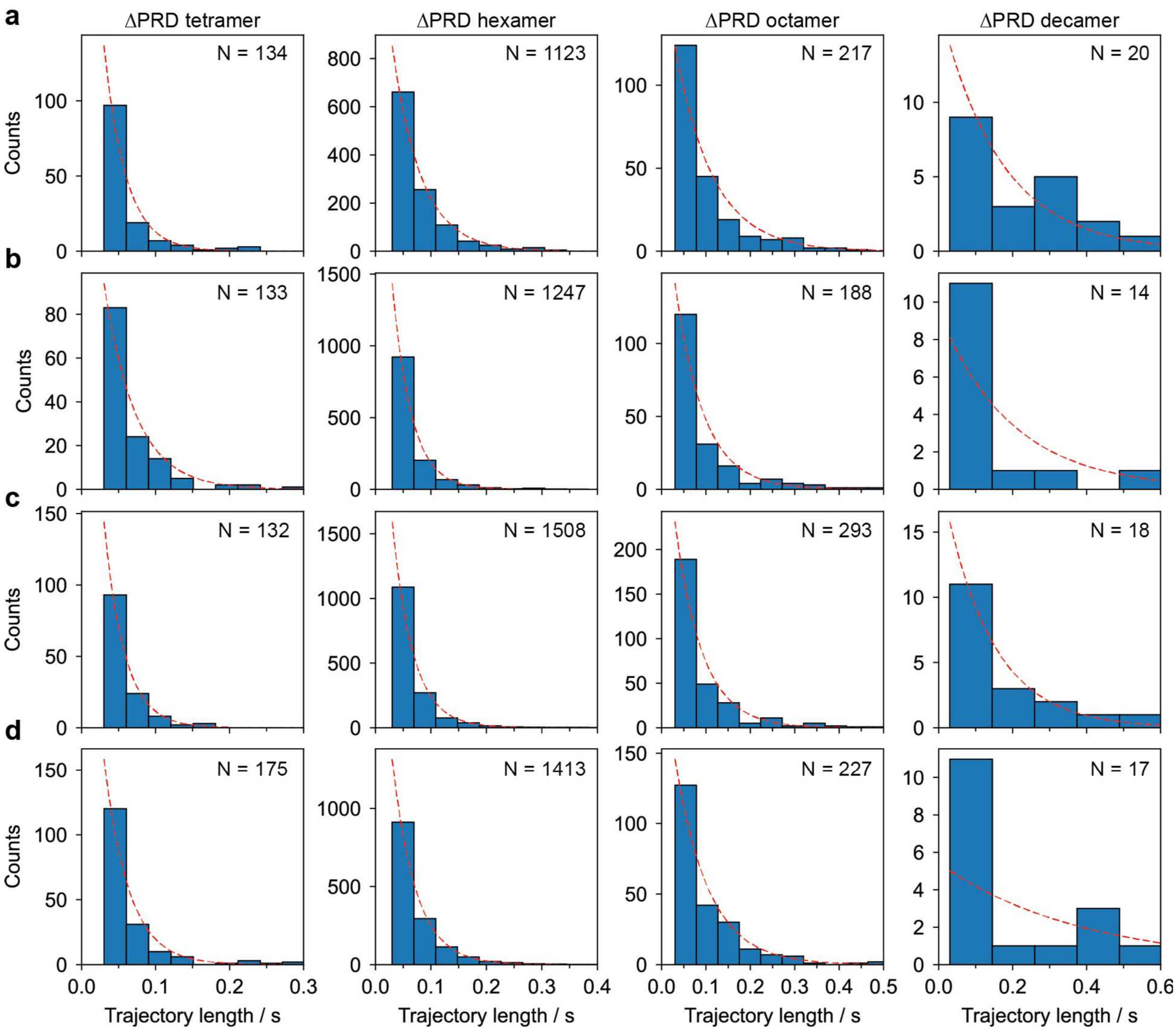

Distribution of trajectory residence times of ΔPRD oligomers on the SLB

(a)-(d) Histograms of counts vs trajectory lengths for each oligomeric species detected in the four repeats of ΔPRD measurements shown in Supplementary Fig. 8. Dissociation rate constants for unbinding from the SLB were calculated by maximum likelihood estimation using the unbinned data (see methods) and the resulting exponential distributions are plotted as red-dashed lines (appropriately scaled for display). The number of decamer trajectories (right column) was small in comparison to the other oligomeric species and thus sometimes resulted in poor fits. Only trajectories that lasted at least 10 frames and contained no gaps were considered.

Supplementary Figure 11

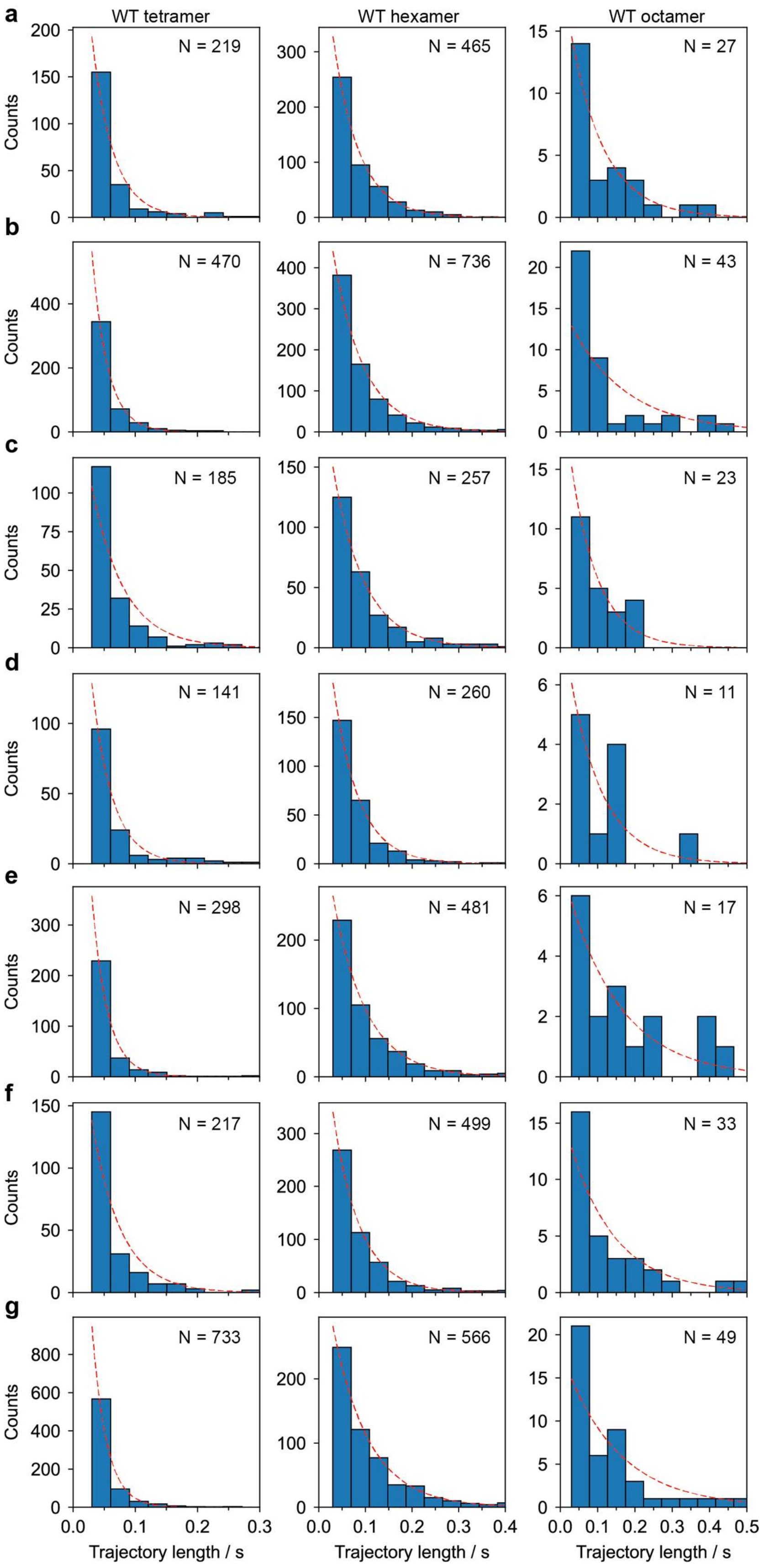

Distribution of trajectory residence times of WT oligomers on the SLB

(a)-(d) Counts vs trajectory length for each oligomeric species detected in the 7 replicate measurements of WT shown in Supplementary Fig. 6. Dissociation rate constants for unbinding from the SLB were calculated by maximum likelihood estimation using the unbinned data (see methods) and the resulting exponential distributions are plotted as red-dashed lines (appropriately scaled for display). The number of octamer trajectories (right column) was small in comparison to the other oligomeric species and thus sometimes resulted in poor fits. Only trajectories that lasted at least 10 frames and contained no gaps were considered.

Supplementary Figure 12

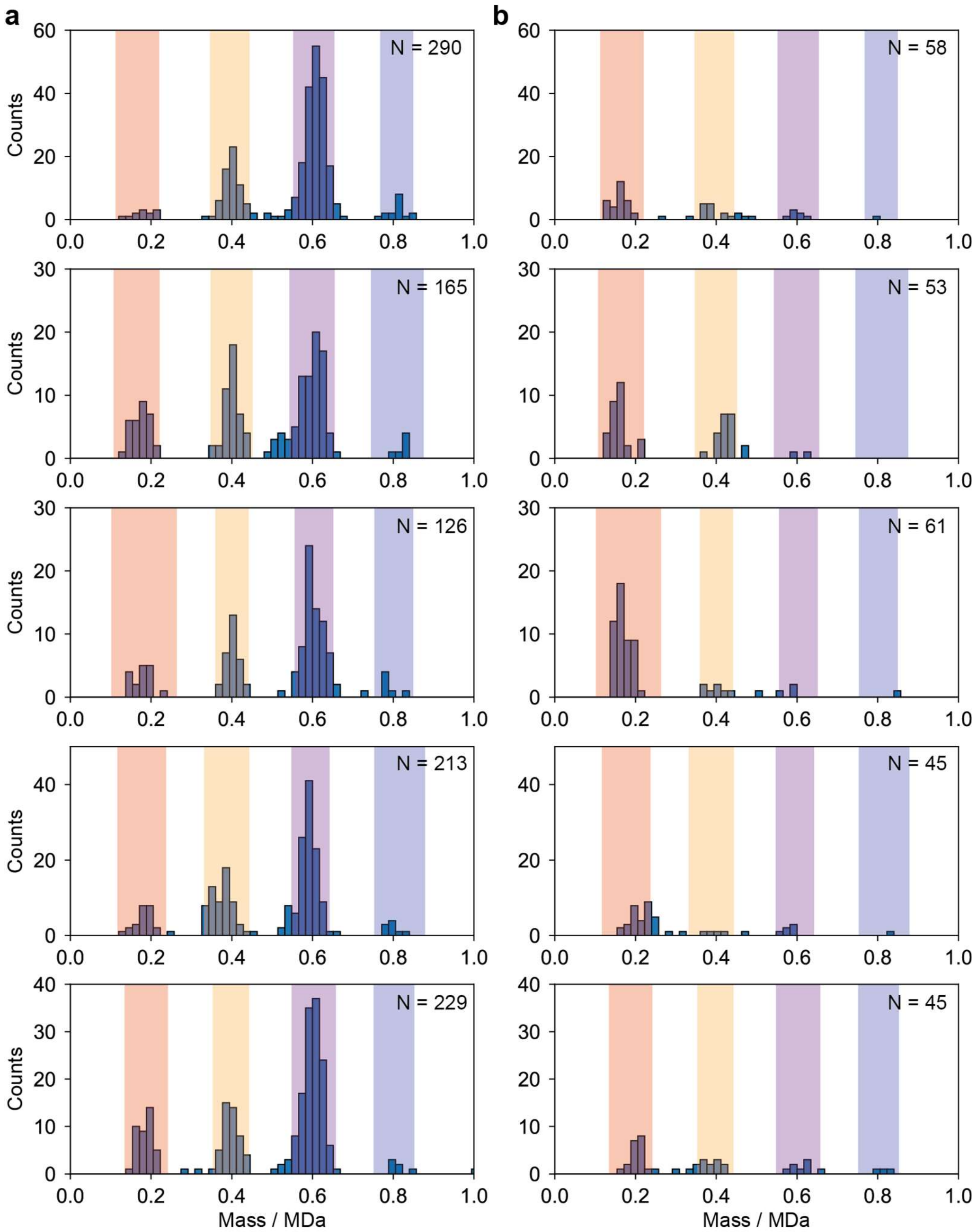

Effect of GTP addition on the mass distribution of WT

Mass distribution of five measurements of WT (10-20 nM) in contact with an SLB before (**a**) and after (**b**) addition of 1 mM GTP (1 min movie each). Shaded areas were used to classify trajectories as dimer (red), tetramer (orange), hexamer (purple) and octamer (blue) as described in the methods section. This data was used to generate the bar plot shown in Fig. 2g. Only trajectories that lasted at least 20 frames were considered.

Supplementary Figure 13

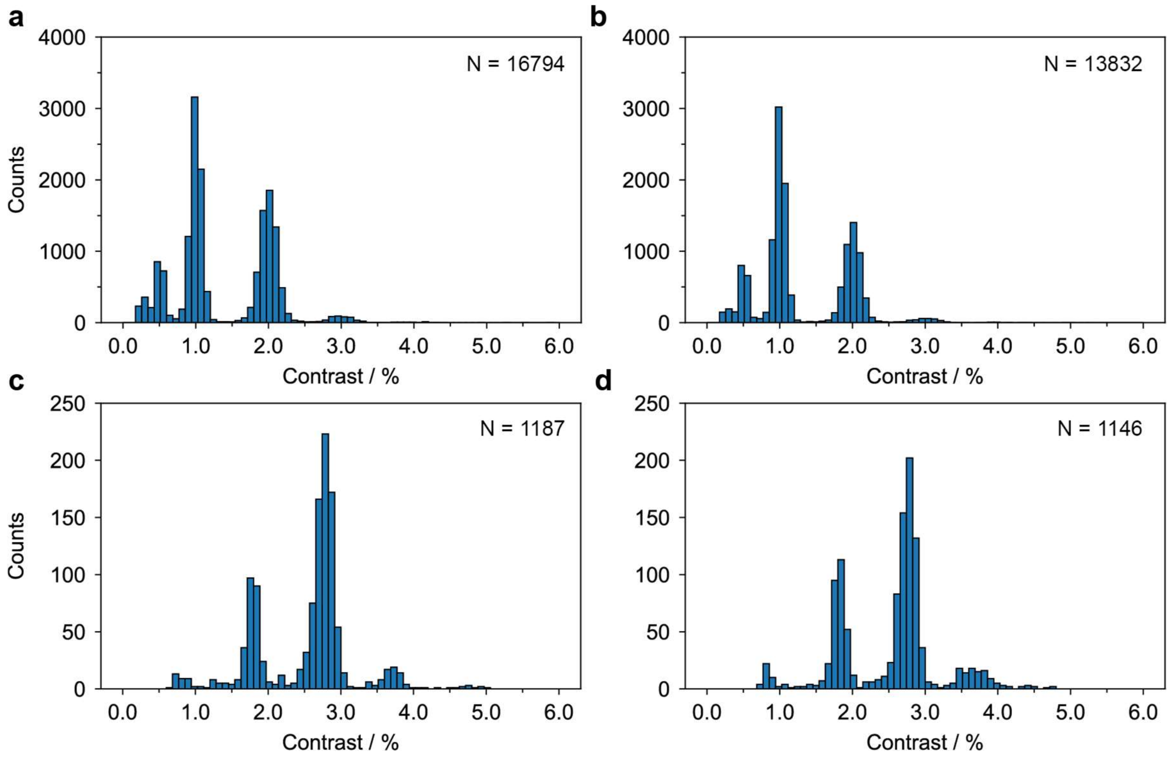

Effect of ultracentrifugation on the measured oligomeric distribution of WT

Contrast histograms obtained from standard MP measurements of 100 nM WT **(a-b)** and dynamic MP measurements of 10 nM WT on an SLB **(c-d)** after centrifugation of the stock solution at 20,000 g **(a, c)** and 100,000 g **(b, d)**. The standard MP histograms consist of four combined repeat measurements (1 min movie each) and the dynamic MP measurements consist of two combined 3 min movies each considering only trajectories that lasted at least 50 frames. The data in (c) is the same as in Extended Fig. 2d.

Supplementary Figure 14

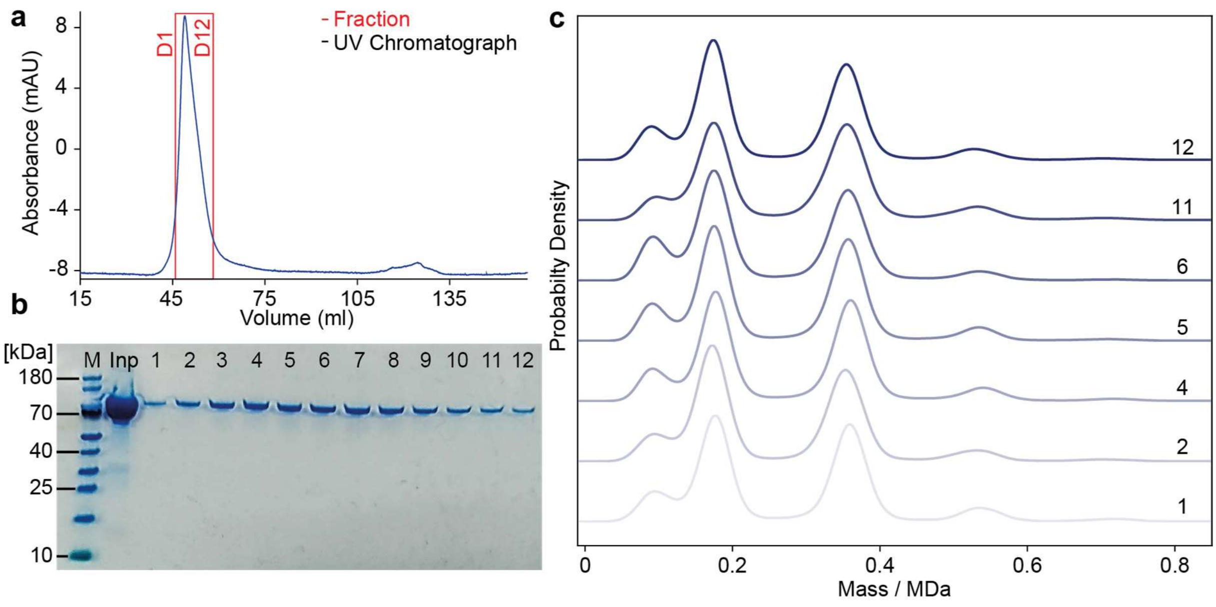

Effect of size exclusion chromatography on the abundance of dynamin oligomers.

(a) Size exclusion chromatography (SEC) elution profile of ΔPRD (13 μM). (**b)** SDS-PAGE of the ΔPRD SEC elution (n = 1 measurement). Input shows the initial concentration subjected to SEC purification. **(c)** Mass distribution of fractions obtained from the SEC elution (all diluted to 200 nM) measured by standard MP. Each histogram is a combination of 4 independent repeat measurements (total particle numbers: N_{D1} = 39012, N_{D2} = 47099, N_{D4} = 70911, N_{D5} = 73538, N_{D6} = 71287, N_{D11} = 73616, N_{D12} = 45450).

Supplementary Figure 15

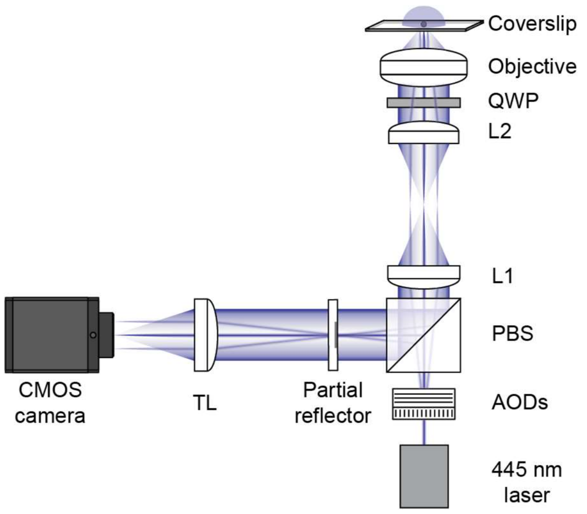

Custom-built setup used in this study

Layout of the custom-built mass photometer used to acquire the data shown in Fig. 1a, e-g and 2e-f, similar to that reported previously[3,4]. A collimated 445 nm laser beam (Lasertack) is directed through an orthogonal pair of acousto-optic deflectors (AODs; AA opto Electronic, DT SXY-400). After passing through a polarising beam splitter (PBS), two telecentric lenses (L1 and L2) direct the deflected beam through a quarter-wave-plate (QWP) and into the back focal plane of the objective (Olympus PlanApo N, 1.42 NA, 60x). This setup results in the beam being weakly focused and scanned across the sample to illuminate a 9.4 x 6.2 μm^2 field of view. The light reflected at the glass-water interface of the coverslip together with the light scattered by the sample is collected by the objective and travels through the same telecentric lens system that the incident light was passed through. Use of the QWP and PBS separates the collected light from the incident light and passes through a partial reflector, which is positioned in the reimaged back focal plane of the objective. This partial reflector is made of a 3.5 mm diameter layer of silver deposited onto a window and attenuates the reflected (at low NA) light by over two orders of magnitude compared to the light scattered by particles[3]. After passing through the partial reflector the light is imaged by a tube lens (TL) onto a CMOS camera (Point Grey, GS3-U3-23S6M-C) resulting in 250x magnification and a pixel size of 23.4 nm. Images are recorded at 1 kHz and then binned in groups of 3 by a custom LabVIEW software that also performs x and y pixel binning (generally 3 x 3) before saving the images for later analysis. Samples are mounted on a custom-built sample stage, with height adjustment provided by a micrometer translation stage (Optosigma) and piezoelectric actuator (Thorlabs AE0505D16F). Sample focus is maintained by an autofocus system (not shown) as follows: a diode laser (Lasertack) is coupled out of a single mode fiber with a 4x objective, and travels collimated into the imaging objective via a dichroic. The beam overfills the objective back aperture and high-NA components are totally internally reflected, causing the back-reflection of an annular beam from the sample. This annular beam is imaged onto a CMOS camera (Thorlabs DCC1545M). Custom labview code continuously measures the radius of this annular beam, and adjusts the piezoelectric actuator in the stage to maintain this radius at a set value. The sample stage and all the optics (except for coupling of the autofocus laser into the single mode fiber) are coupled to a 600x400x50mm aluminium breadboard, enclosed by 40mm thick aluminium walls and lid, which sits on a granite table.

Supplementary Figure 16

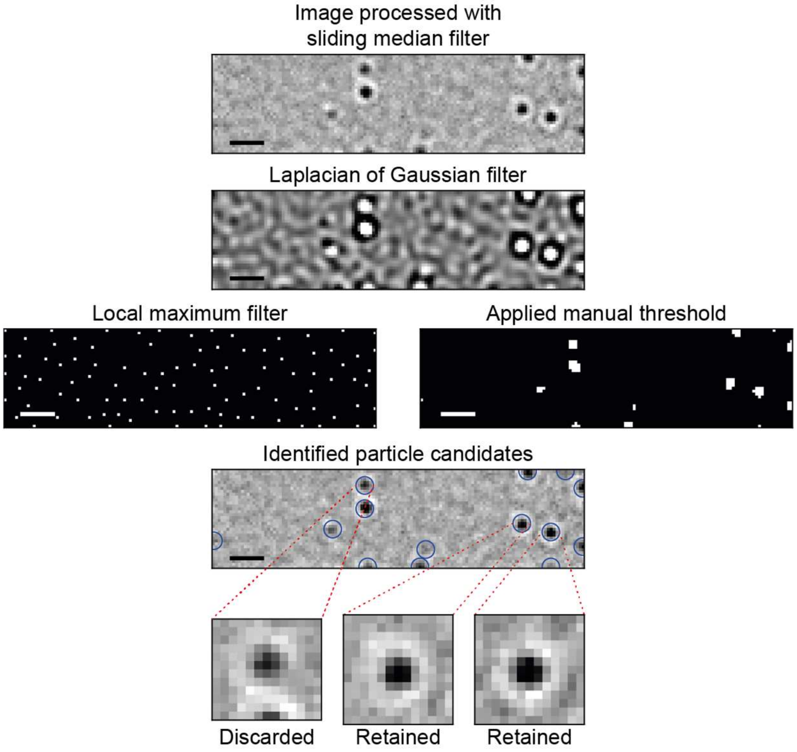

Image processing for identification of particle candidates

Background-subtracted images are filtered with a Laplacian of Gaussian kernel with a size to match the size of PSFs in the images (sigma = 1.5). Next, a manually set threshold and local maximum filter are applied to the filtered image. By combining the resulting binary maps, we obtain single pixels at the centre of potential parPticle candidates (circled in blue). From each of these pixels a 13x13 region of interest is constructed to which our PSF model is then applied for quantification of particle contrast and position. If the pixel candidate is too close to an edge of the field of view to construct a complete 13x13 region of interest, it is discarded, as shown in the example here. Scale bar = 1 μm. The images shown in this figure were obtained by applying our image processing pipeline to a snapshot from one dynamic MP movie. This approach produced similar results in all dynamic MP movies used in this study (n > 30).

Supplementary Figure 17

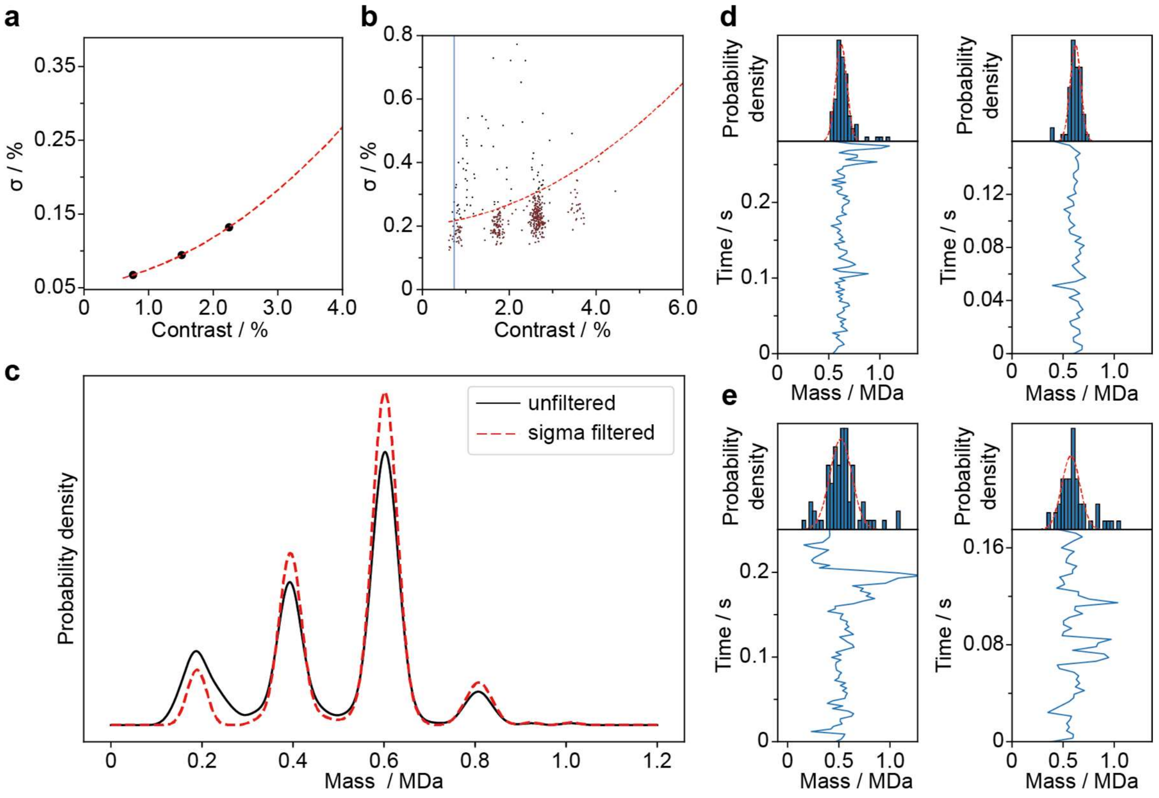

Filtering of trajectories using the standard deviation of their contrast distributions

(a) Standard deviation (σ) vs mass for WT oligomers obtained from standard MP experiments in solution. **(b)** Standard deviation vs mass scatter plot for the trajectories obtained from the dynamic MP measurement of WT shown in Fig. 1b-d and 2a (after length filtering). The solution-phase trend from (a) is applied with an appropriate offset as a threshold (here 0.15%). Trajectories below the threshold (red) are used for analysis. In this particular case, trajectories with a standard deviation in mass below 170 kDa (blue line) were not used to reduce the number of trajectories that likely result from background noise in the analysis. **(c)** Effect of the applied threshold on the mass distribution of trajectories. **(d)** Examples of trajectories that passed filtering. **(e)** Examples of trajectories that did not pass filtering.

Supplementary Figure 18

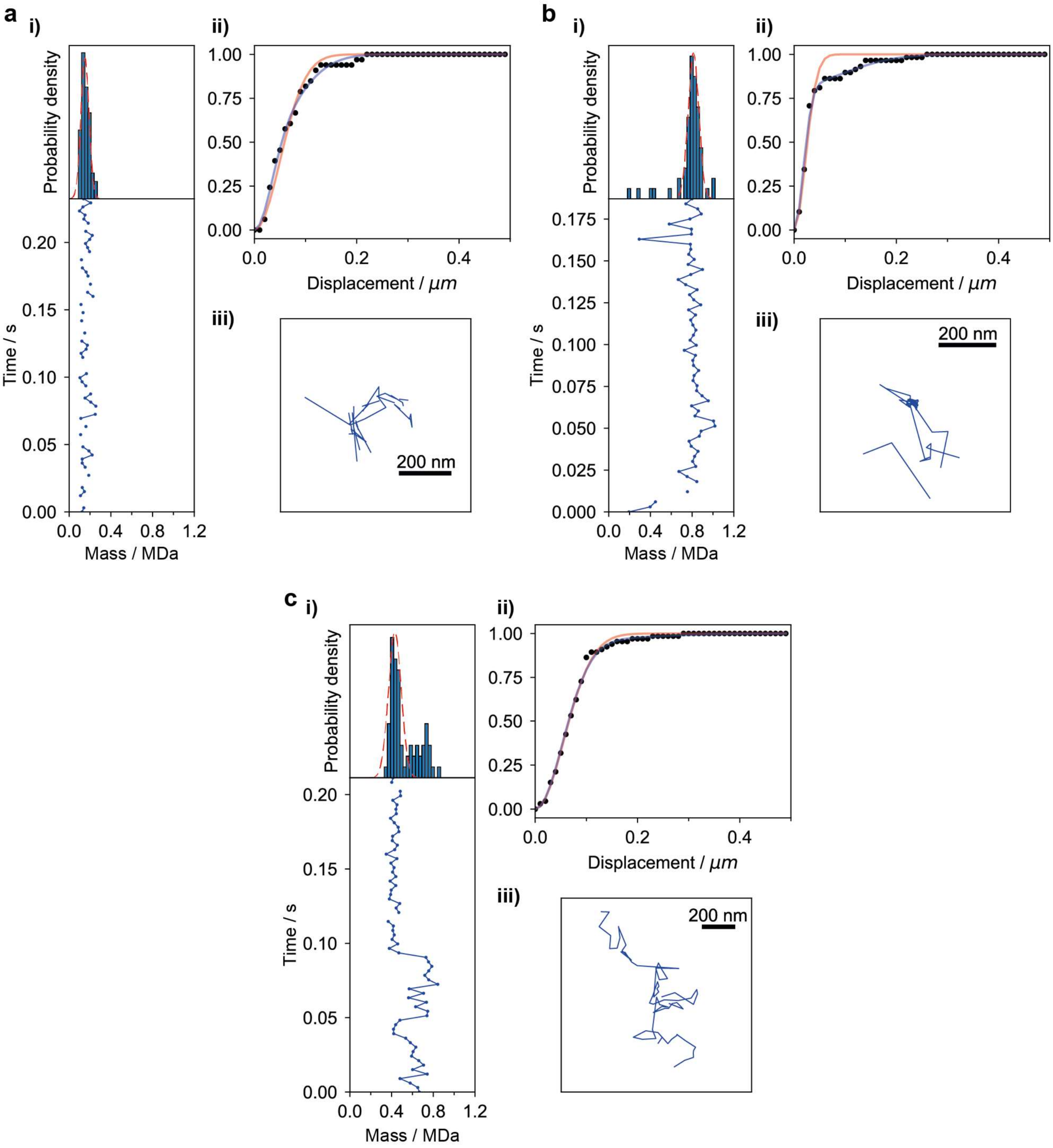

Examples of particles that were excluded from the diffusion analysis

(a) Mass trace (**i**), cumulative probability density distribution of 1-frame displacement with 1-and 2-component mobility fits in red and blue, respectively (**ii**), and trajectory in 2D space (**iii**) of a particle that was excluded from the diffusion analysis because it was above the set threshold of allowed trajectory gaps. **(b**) Same as (a) but for a particle that was excluded because it was determined to be too stationary during its time on the SLB. **(c)** same as (a) but for a particle with a mean mass that deviated too far from the mass determined by Gaussian fitting (red dashed line), which was usually a sign of an incorrectly linked trajectory. These examples of excluded particles were chosen from the data shown in Fig. 1b-c and 2a.

Supplementary Tables

Supplementary Table 1. Mean contrast values ± standard deviation of WT measured with standard MP (MP; n = 3 independent measurements) and dynamic MP (DMP; n = 6 independent measurements)

Oligomeric species	MP contrast / %	DMP contrast / %
Dimer	1.00 ± 0.01	0.83 ± 0.05‡
Tetramer	2.01 ± 0.01	1.82 ± 0.01
Hexamer	3.03 ± 0.02	2.79 ± 0.02
Octamer	4.03†	3.74 ± 0.04

†Extrapolated from calibration slope
‡Partially overlapped with background noise

Supplementary Table 2. Mean contrast values and standard deviations (in %) of trajectories obtained from a movie generated by simulating randomly diffusing point spread functions representative of oligomeric species of WT dynamin at different particle densities onto a dynamic MP movie of HKS-100 buffer in contact with a supported lipid bilayer (n = 1 simulated movie for each particle density). One set of simulations included simulating particle movement during image acquisition ('blurred') and one set did not simulate this effect ('static', *i.e.* particle movement occurs exactly in between frames). Point spread functions were simulated onto the raw images with contrast values of 1.00% (dimer), 2.00% (tetramer), 3.00% (hexamer) and 4.00% (octamer).

Particle density / μm^{-2}	Dimer (static)	Dimer (blurred)	Tetramer (static)	Tetramer (blurred)	Hexamer (static)	Hexamer (blurred)	Octamer (static)	Octamer (blurred)
0.13	1.01 ± 0.06	0.90 ± 0.05	2.00 ± 0.06	1.83 ± 0.06	2.98 ± 0.06	2.81 ± 0.07	3.97 ± 0.07	3.77 ± 0.07
0.30	1.01 ± 0.06	0.90 ± 0.07	2.00 ± 0.06	1.84 ± 0.07	2.99 ± 0.07	2.80 ± 0.07	3.98 ± 0.07	3.77 ± 0.08
0.79	1.03 ± 0.07	0.90 ± 0.08	2.01 ± 0.08	1.86 ± 0.09	3.00 ± 0.09	2.82 ± 0.10	3.99 ± 0.09	3.78 ± 0.11

Supplementary Table 3. Mean contrast vs mass calibration slopes ± standard deviation obtained from the simulated dynamic MP movies shown in Supplementary Fig. 6 b,e (at 0.30 μm^{-2} particle density, n = 1 movie for 'static' and 'blurred') and from n = 3 standard MP and n = 6 dynamic MP measurements of WT dynamin (Extended Data Fig. 2c-d).

	Simulated data (static)	Simulated data (blurred)	Standard MP	Dynamic MP
Contrast vs mass slope / % MDa^{-1}	4.99 ± 0.01	4.68 ± 0.03	5.04 ± 0.01	4.63 ± 0.06

Supplementary Table 4. Mean diffusion coefficients (D), dissociation constants from the SLB (k) and mean trajectory lengths for WT dynamin (n = 7 repeat measurements; Extended Fig. 5, 7). Mean trajectory lengths were calculated from the dissociation constant of each species (mean = 1/k for one component exponential distributions) to avoid bias towards shorter trajectories (< 10 frames) due to background features being identified as particles.

	Tetramer (0.40 MDa)	Hexamer (0.60 MDa)	Octamer (0.80 MDa)
D / μm^2 s^{-1}	0.81 ± 0.05	0.61 ± 0.03	0.50 ± 0.04
k / s^{-1}	32 ± 9	16 ± 2	9 ± 3
Mean trajectory length / s	0.03 ± 0.01	0.06 ± 0.01	0.11 ± 0.03

Supplementary Table 5. Mean diffusion coefficients (D), dissociation constants from the SLB (k) and mean trajectory lengths of ΔPRD (n = 4 repeat measurements; Fig. 2b, d). Mean trajectory lengths were calculated from the dissociation constant of each species (mean = 1/k for one component exponential distributions) to avoid bias towards shorter trajectories (< 10 frames) due to background features being identified as particles.

	Tetramer (0.36 MDa)	Hexamer (0.54 MDa)	Octamer (0.72 MDa)	Decamer (0.90 MDa)
D / μm^2 s^{-1}	0.82 ± 0.05	0.67 ± 0.02	0.52 ± 0.02	0.46 ± 0.03
k / s^{-1}	31 ± 5	25 ± 4	14 ± 2	5 ± 2
Mean trajectory length / s	0.03 ± 0.01	0.04 ± 0.01	0.07 ± 0.01	0.19 ± 0.07

References

1. Dar, S., Kamerkar, S. C. & Pucadyil, T. J. A high-throughput platform for real-time analysis of membrane fission reactions reveals dynamin function. *Nat. Cell Biol.* **17**, 1588–1596 (2015).

2. Deo, R. *et al.* ATP-dependent membrane remodeling links EHD1 functions to endocytic recycling. *Nat. Commun.* **9**, 5187 (2018).

3. Cole, D., Young, G., Weigel, A., Sebesta, A. & Kukura, P. Label-Free Single-Molecule Imaging with Numerical-Aperture-Shaped Interferometric Scattering Microscopy. *ACS Photonics* **4**, 211–216 (2017).

4. Young, G. *et al.* Quantitative mass imaging of single biological macromolecules. *Science* **360**, 423–427 (2018).
